# The prevalence of chronic traumatic encephalopathy in a historical epilepsy post‐mortem collection

**DOI:** 10.1111/bpa.13317

**Published:** 2024-11-11

**Authors:** Maritchka Ryniejska, Hanaa El‐Hachami, Alicja Mrzyglod, Joan Liu, Maria Thom

**Affiliations:** ^1^ Department of Clinical and Experimental Epilepsy University College London (UCL) Queen Square Institute of Neurology London UK; ^2^ School of Life Sciences University of Westminster London UK

**Keywords:** astrocytopathy, chronic traumatic encephalopathy, epilepsy

## Abstract

Previous post‐mortem epilepsy series showed phosphorylated tau (pTau) accumulation in relation to traumatic brain injury (TBI) rather than driven by seizure frequency. The Corsellis Epilepsy Collection, established in the mid‐20th century, represents brain samples collected from patients living with a range of epilepsies from the 1880s to 1990s. Our aim was to interrogate this historical archive to explore relationships between epilepsy, trauma and tau pathology. AT8 immunohistochemistry for pTau was carried out in 102 cases (55% male, with mean age at death of 62 years) on frontal, temporal, amygdala, hippocampal and lesional cortical regions and evaluated using current NINDS criteria for chronic traumatic encephalopathy (CTE) and Braak staging with beta‐amyloid, AT8‐GFAP and other pTau markers (CP13, PHF1, AT100, AT180) in selected cases. CTE‐neuropathologic change (CTE‐NC) was identified in 15.7% and was associated with the presence of astroglial tau, a younger age of onset of epilepsy, evidence of TBI and institutionalisation for epilepsy compared to cases without CTE‐NC, but not for seizure type or frequency. Memory impairment was noted in 43% of cases with CTE‐NC, and a significantly younger age of death; more frequent reports of sudden and unexpected death (*p* <0.05–0.001) were noted in cases with CTE‐NC. In contrast, a higher Braak stage was associated with late‐onset epilepsy and cognitive decline. Of note, 9% of cases showed no pTau, including cases with long epilepsy duration, poor seizure control and a history of prior TBI. In summary, this cohort includes patients with more severe and diverse forms of epilepsy, with CTE‐NC observed more frequently than reported in non‐epilepsy community‐based studies (0%–8%) but lower than published series from contact sports participants (32%–87%). Although the literature does not report increased epilepsy occurring in CTE syndrome, our findings support an increased risk of CTE in epilepsy syndromes, likely primarily related to increased TBI.

## Graphical abstract

In an archival epilepsy post‐mortem collection from last century, chronic traumatic encephalopathy identified in 15.7% was associated with traumatic brain injury. Alzheimer's disease neuropathology change was increased in late‐onset epilepsy but in 8% of cases with severe epilepsy there was no tau pathology found.

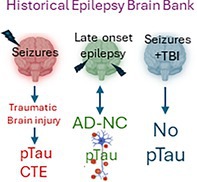

## 1 INTRODUCTION

There is ongoing interest and controversy surrounding the clinical significance of tau protein accumulation in patients with epilepsy. Tau promotes epileptogenesis in diverse animal models [1]; the most compelling evidence comes from studies which demonstrated that reduction of Tau load reduced excitability, seizures and normalised hippocampal networks [2, 3, 4]. Focal accumulation of phosphorylated tau (pTau) has been variably reported in epilepsy surgical resections [5, 6]. There is a bidirectional relationship between Alzheimer's disease (AD) and late‐onset epilepsy (LOE) based on shared pathology and overlapping risk factors. Both conditions are associated with pathological tau (ptau), neuronal loss, white matter degeneration and neurovascular pathology [7]. However, memory dysfunction in epilepsy is not just related to tau pathology, but likely to be more complex and multifactorial [8].

Post‐mortem series in epilepsy provides an opportunity to study the extent, patterns and distribution of any abnormal tau accumulation following a lifetime of seizures and to draw comparisons with other tauopathies. In our previous published post‐mortem series collected between 1988 and 2009, a key finding was that increased pTau accumulation, including astrocytic tau, was associated with a history of brain trauma rather than frequency of seizures and duration of epilepsy. However, pTau was not the sole explanation for the cognitive decline [9]. The pathological stages and patterns of tau accumulation following repetitive brain injury, mainly from sports injuries, have since been extensively characterised as ‘chronic traumatic encephalopathy’ (CTE) [10, 11, 12], and in epilepsy, CTE has been reported at post‐mortem in isolated poorly controlled cases [13, 14] and CTE neuropathological change (CTE‐NC) was also noted in samples taken during epilepsy surgery [15, 16].

Based on observations from the Corsellis Brain Collection, the foremost and one of the largest brain collections of the last century, degenerative brain changes following trauma due to boxing were originally outlined in a study by Corsellis et al. [17]. This tissue has since been re‐evaluated with modern immunohistochemistry to successfully confirm CTE pathology in a proportion [18]. The Corsellis brain collection was also the basis for many seminal publications on the ‘aftermath of epilepsy’ and cellular effects of seizures [19, 20, 21]. As this brain collection includes cases with varied epilepsy syndromes and severity from patients alive in the 19th–20th centuries, many in epilepsy care institutions, it provides a unique opportunity to investigate the ‘natural history’ of tau pathology in people naïve to modern epilepsy treatments, in comparison with more current epilepsy series. We hypothesised that CTE‐NC would be more prevalent in Corsellis epilepsy cases with more poorly controlled seizures or histories of head injury, either as a result of or cause of seizures.

## 2 METHODS

### 2.1 Ethics approval and consent

The project is approved by the Epilepsy Society Brain and Tissue Bank (NHS Health Research Authority, Research Ethics Committee Reference number 23/SC/0002) and use of patient samples and data in research from this archive adheres to the Human Tissue Act of the United Kingdom (2004).

### 2.2 Case selection criteria

The ERUK Corsellis Epilepsy Collection (CEC) within UCL Epilepsy Society Brain and Tissue Bank, comprises a collection of brain tissue samples of 378 surgical and post‐mortem cases from patients living between 1890s and 1990s. All original formalin‐fixed paraffin‐embedded (FFPE) blocks were restored, re‐sectioned and reviewed, together with original histology slides with varied tinctorial stains, macroscopic brain images and available linked clinical data. We excluded surgical samples, cases with primary high grade CNS tumours, age less than 25 years at death, limited clinical data or an unclear epilepsy history (e.g. symptomatic terminal and alcohol withdrawal seizures) and suboptimal tissue samples.

From selected cases, regions of the frontal cortex (or other cortical regions if frontal cortex not available), temporal cortex, hippocampus (all hippocampal blocks if hippocampal sclerosis was present), amygdala and any cortical focal brain lesion were included (see Table S1 for regions analysed in each case). The post‐mortem interval (PMI), fixation time and age of the FFPE blocks were recorded.

#### 2.2.1 Immunostaining and evaluation

pTau immunostaining was carried out with monoclonal Antibody AT8 (Ser202, Thr205), an epitope resistant to long tissue fixation (Pikkarainen et al.) [22], on five‐micron FFPE sections. Beta‐amyloid staining was carried out on 64 cases with high tau load and GFAP‐AT8 chromogenic double labelling in 14 cases with prominent astroglial tau. In three randomly selected cases with CTE‐NC patterns, additional pTau phosphorylation sites were investigated with PHF1 (Ser396, Ser404), AT100 (Thr212, Ser214), AT180 (Thr231) and CP13 (Ser202) to enable comparison with previous published studies (see Data S1 for staining protocols). All slides were scanned on the Hamamatsu digital slide scanner (Hamamatsu 360, Hamamatsu Photonics) at ×40 magnification and assessed independently by two observers (MT, MR). Immunofluorescence sections were scanned with a Hamamatsu NanoZoomer S60 Digital slide scanner (C13210‐04, Hamamatsu Photonics).

Braak staging was estimated based on the mesial temporal and cortical regions available; in cases with no or partial hippocampal subfield representation, the case was not staged. For CTE staging, we employed NINDS/NIBIB criteria for the identification of pathognomonic lesions, comprising pTau perivascular aggregates in neurones, with or without astrocytes, at the depth of a sulcus and not restricted to the subpial region [13]. We noted how many cortical regions were involved and if typical hippocampal CTE pathology was evident. We also noted cases with suggestive features of CTE‐NC, but which fell short of definitive criteria, for example, prominent subpial sulcal pTau.

#### 2.2.2 Clinical data

The clinical data were extracted from the available records, including documentation of head or bodily injuries sustained because of a seizure or otherwise, medications, surgical treatments, developmental delay or memory decline and if patients resided in an epilepsy care home or equivalent setting. Together with the original neuropathology slides and reports, the likely cause of epilepsy and the cause of death were categorised where possible.

#### 2.2.3 Statistical analysis

Fisher's exact test was used to compare categorical variables and a logistic regression analysis between pathology groups with or without CTE‐NC (SPSS version 28, IBM), with *p* values of 0.05 or lower taken as significant. The Mann–Whitney non‐parametric test was used for Braak staging between cohorts and GraphPad Prism version 9 was used for graphical representations.

## 3 RESULTS

### 3.1 Epilepsy history

The selected CEC series comprised 102 cases, 55% male, born between 1884 and 1962 (median birth year 1919), and post‐mortem examinations were conducted between 1965 and 1996, with a mean age at death of 61.6 (range 25–89) years (Table S1 for case detail). Fifty‐nine patients had been resident in an institution, care home or ‘epilepsy colony’ for some period of their life, including the Chalfont Centre for Epilepsy. The age of onset was available in 91 cases (mean 30.7 years, range 0.1–84) which we categorised as either early‐onset epilepsies (EOE <20 years [*n* = 48], including 31 with age of onset <10 years), adult‐onset epilepsies (AOE 21–40 years; *n* = 14) or late‐onset epilepsy (LOE >40 years; *n* = 29). Information on the duration of epilepsy was available in 70 cases (mean 26.8 years, range 0.1–71 years) categorised as either short‐term (<2 years, *n* = 13), medium‐term (2–10 years, *n* = 11) and long‐term epilepsy (>11 years, *n* = 72). Seizure types were classified as generalised, including descriptions such as ‘grand‐mal’, ‘major’ or tonic–clonic seizures (*n* = 78) or focal seizures reported as ‘petit mal’, complex partial seizures or ‘minor’ seizures (*n* = 36), with no information on seizure type available in 12 cases. Other main seizure types and events recorded included episodes of status epilepticus (*n* = 13) and absence seizures (*n* = 4). Information on seizure control, such as seizure diaries, was available in 68 cases with details of prescribed anti‐seizure medications (ASM) in 58 cases, mainly phenobarbitone, phenytoin, carbamazepine and sodium valproate (Table S1). Seizure frequency or refractoriness was categorised as ‘well‐controlled’ with rare seizures reported, including where ASM were stopped (*n* = 20), ‘refractory’ with frequent generalised seizures, episodes of poor control, seizure clusters or episodes of status epilepticus (*n* = 20) and moderate seizure control (*n* = 28). In 34 cases, it was not possible to categorise seizure refractoriness due to limited information. Scalp EEG reports were available in a subset of patients but not systematically recorded and not further evaluated for the purposes of this study. Limited neuroimaging reports were available and no MRI studies.

### 3.2 Co‐morbidities, history of head injury

In 18 cases, there was clear documentation of an early developmental delay or deficit, including learning disability, with the use of outdated terminologies such as ‘imbecile’, ‘idiot’, 'subnormal’ and ‘backward’. In 31 cases there was a record of memory decline or dementia during the illness course. Information on falls, injuries and fractures was documented in the notes in 30 cases, although not always specified if related to a seizure event. In 18 cases, there was documentation of a head injury during life, for example, scalp laceration, bruising or skull fracture.

### 3.3 Aetiology of epilepsy and cause of death

The weight of the brain was available in all cases, (mean, 1228 g; range, 702–1632 g) (Table S1). In 71 cases, at least one focal pathology was identified, including hippocampal sclerosis (HS, *n* = 29; unilateral ILAE type 1 [*n* = 16], unilateral ILAE type 2/3 [*n* = 3], bilateral ILAE type 1 [*n* = 3], bilateral asymmetrical HS [*n* = 7]), malformations of cortical development, including polymicrogyria and heterotopia (*n* = 8) and perinatal infarcts/ulegyria (*n* = 5). Other acquired neuropathology included old TBI, mainly contusions (*n* = 23), cerebrovascular disease (*n* = 40; grouped as regional chronic infarct [*n* = 8], lacunar infarct/microinfarcts/significant small vessel disease [*n* = 27], subacute infarction/haemorrhage [*n* = 5]) and neuroinflammatory disease (*n* = 3, sarcoidosis, SLE‐related vascular disease). Cerebellar atrophy was also frequently reported but not further categorised for this study. Following the review of the neuropathology findings and clinical data, the cases could be classed according to the likely aetiology of the epilepsy in the majority of cases (Table S2). Of note, seizures documented following a TBI and likely post‐traumatic epilepsy (PTE) were noted in 6 cases.

The cause of death was recorded in the post‐mortem records in 100 cases. The recognition and terminology for sudden and unexpected death in epilepsy (SUDEP) was not used during the era of this brain collection. Cases with a witnessed death during a seizure or nocturnal sudden and unexpected death for whom no clear cause of death was identified at post‐mortem examination (toxicology reports were mainly not available) were re‐classified as probable SUDEP (*n* = 17). Deaths due to status epilepticus or accidental deaths were classed as ‘other epilepsy‐related deaths’ (*n* = 6) and the remainder as non‐epilepsy‐related deaths (*n* = 77) (Table S1). The post‐mortem interval was available in 78 cases (mean 2 days; range 0.5–7). Fixation times were available in 44 cases (mean 120 days; range 20–1000), and the mean age of the FFPE tissue blocks was 41 years (range 26–57).

### 3.4 Evaluation of tau labelling

**FIGURE 1:**
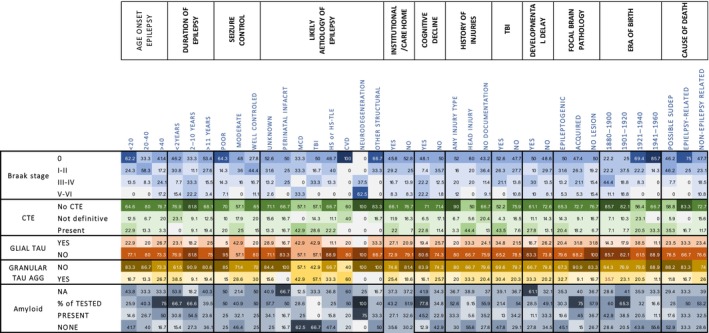
A heat map summary of tau patterns in the Corsellis Epilepsy series in relation to clinical factors. Summary data are presented for Braak staging, CTE, glial tau, granular aggregates and beta‐amyloid showing the percentage of cases with pathology in relation to epilepsy and clinical history with darker colours representing higher percentages. CTE, chronic traumatic encephalopathy‐neuropathology change; CVD, cerebrovascular disease; HS, hippocampal sclerosis; MCD, malformation of cortical development; NA, not assessed; SUDEP, sudden and unexpected death in epilepsy; TBI, traumatic brain injury.

The brain regions evaluated for AT8 labelling varied from 1 to 9 per case with a mean number of three regions, including hippocampal blocks available in 80 (1–4 blocks/case), amygdala in 33, cortical regions (temporal, frontal, occipital, parietal or insular) in 93 (1–4 blocks/case) and in 27 cases a cortical block with a focal lesion (e.g. contusion, infarct and malformation). The findings in relation to clinical variables and pathology factors are summarised in Figure 1 and detailed below.

#### 3.4.1 CTE‐NC pathology

**FIGURE 2:**
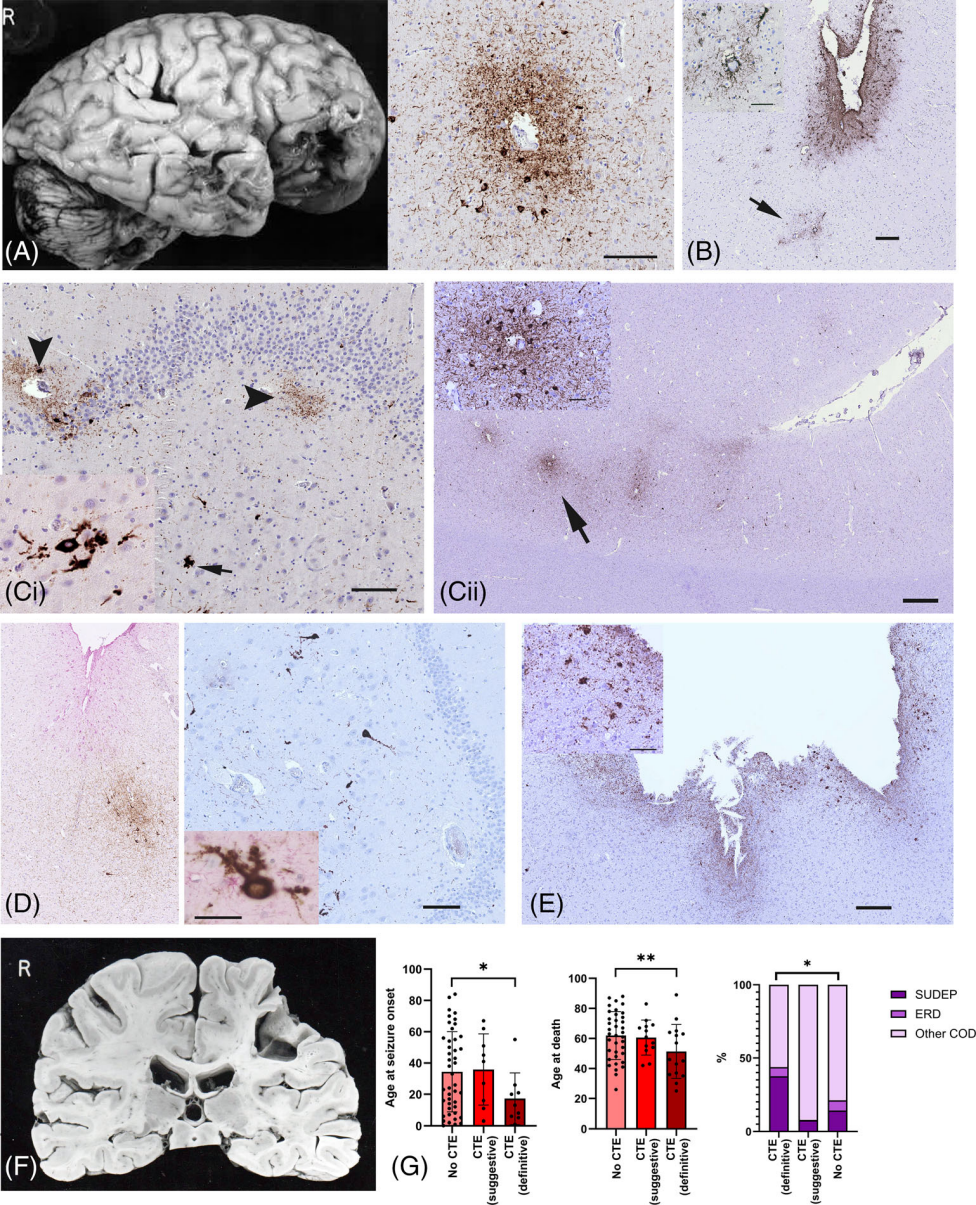
Chronic traumatic encephalopathy‐neuropathology change (CTE‐NC) and tau pathology on AT8 staining, examples from the Corsellis Epilepsy Collection. (A) (CC 9‐68). A 62 male resident of the Chalfont centre for epilepsy with onset of generalised seizures at the age of 20 with no established underlying cause, frequent head injuries including a skull fracture and poorly controlled seizures despite three anti‐seizure medications. He had a witnessed sudden death following a run of seizures with no cause of death found at post‐mortem (possible SUDEP). There were old contusions at post‐mortem examination and a focus of perivascular neuronal tau in the superior temporal gyrus (Bar = 100 microns). (B) (CC44‐95). A 65‐year‐old male with onset of seizures at age 55 following a road traffic accident. Seizures were infrequent and this was resumed post‐traumatic epilepsy. He died of a pulmonary embolus and brain examination confirmed old contusions with tau in neurones and glia at the contusion site as well as two other cortical regions with sulcal and perivascular foci (region arrowed shown in inset). Bar = 200 microns in main image and 100 microns in inset). (Ci) (CC232‐72). A 36‐year‐old male with onset of epilepsy at age 6 following head trauma and presumed post‐traumatic epilepsy; seizures were poorly controlled with up to 260 seizures/year reported. There was also a record of boxing and winning trophies as well as reports of frequent injuries and cognitive decline. The cause of death was given as bronchopneumonia. Old brain contusions were identified, cortical sulcal perivascular tau and in the hippocampus shown, AT8 labelling was observed in granule cells, with granular aggregates, some near vessels (arrowheads) and scattered CA4 ‘mossy’ cells (arrow and cells shown at higher magnification in inset) but not in other hippocampal subfields and minimal staining in the entorhinal cortex. Bar = 100 microns. (Cii) (CC114‐78) A 57‐year‐old male with seizure onset age 13 of unknown cause and resident in an epilepsy care home (Chalfont Centre). There was no history of head trauma, but memory decline reported and contusions and hydrocephalus were confirmed at post‐mortem with two foci of sulcal, perivascular tau (arrow, shown in inset, bar = 50 microns and 500 microns in main image). The cause of death was choking on a food bolus secondary to a pseudobulbar palsy. (D) (CC48‐87) A 37‐year‐old male with onset of epilepsy age 8, resident in an epilepsy centre and with no history of head trauma or brain injury confirmed at post‐mortem and a suspected SUDEP. CTE lesions were present in three cortical regions in sulci (AT8/GFAP stain) and CA4 neurones (bar 100 microns) (inset GFAP/AT8 stain, bar = 50 microns). (E) (CC48‐92) A 62‐year‐old female with age of onset of epilepsy 26 years. There were documented falls and dementia in life and contusions which were confirmed at post‐mortem. AT8 showed positive labelling of astroglia (shown in inset) in wall of a temporal lobe contusion, and she had mixed CTE and AD‐NC. (Bar in main figure 200 microns and 100 microns in inset). (F) (CC112‐75). An 87‐year‐old male who sustained a penetrating brain injury (grenade during WW1) and subsequent seizures although limited information on their frequency. There were few cortical samples available but no tau pathology. (G) Scatter graphs of the age of seizure onset (where recorded) in relation to the presence of CTE pathology (compared to cases with CTE and cases with suggestive but not definitive CTE pathology) with a significantly lower age in this group and younger age at death; bar chart showing the percentage of cases with possible SUDEP was significantly higher in the CTE group than cases without CTE. *p <0.05; **p <0.01 (see Table 1). AD‐NC, Alzheimer's disease neuropathology change; TBI, Traumatic brain injury; SUDEP, Sudden and unexpected death in epilepsy.

**FIGURE 3:**
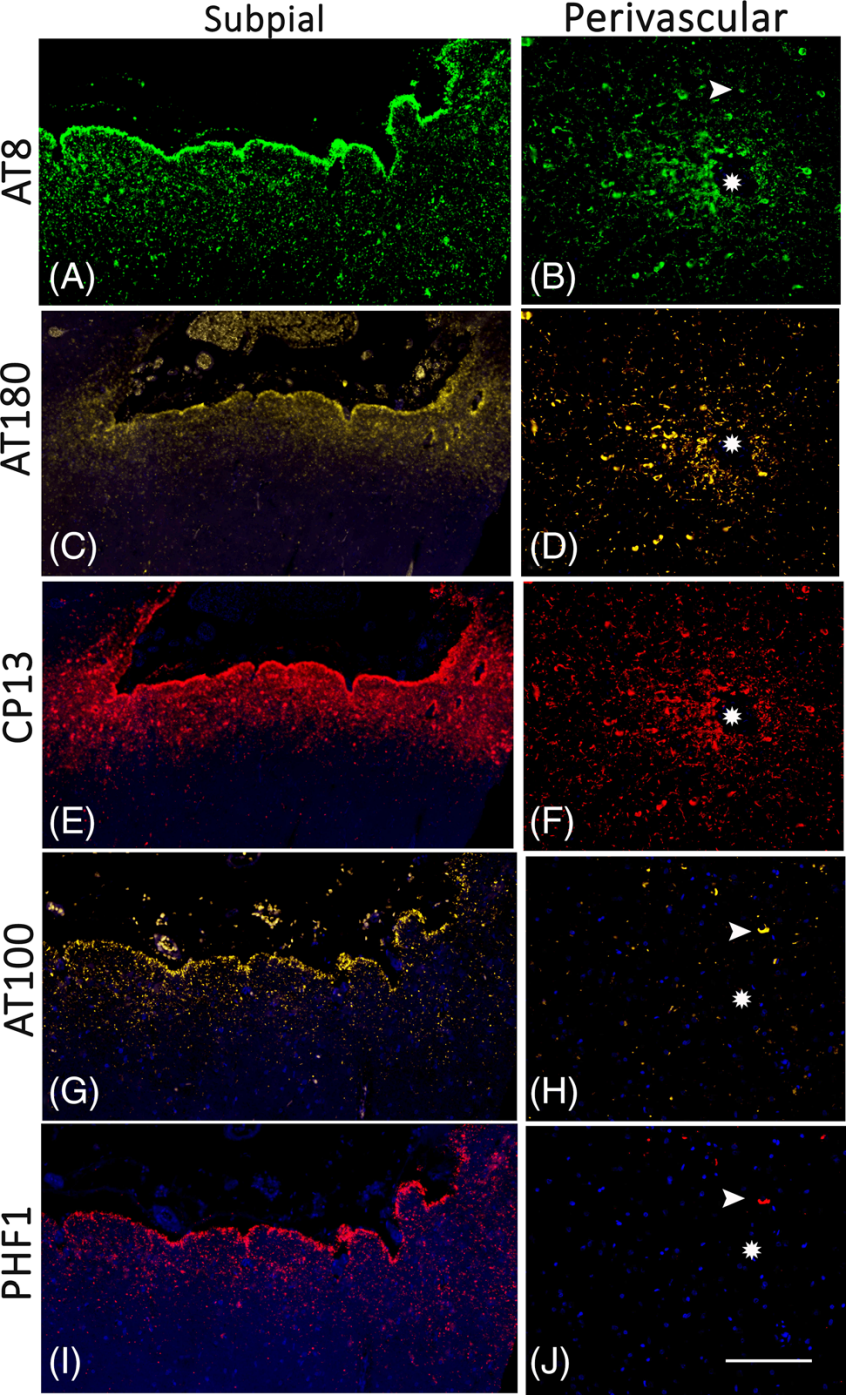
Immunofluorescence of phosphorylated tau epitopes in chronic traumatic encephalopathy‐neuropathology change (CTE‐NC) lesions. (A, C, E, G and I) (CC121‐78) Subpial and layer I astrocytic tau with strong and equivalent labelling of glia for AT8 (A) and CP13 (E). Reduced labelling was noted with AT180 (C), AT100 (G) and PHF1 antibodies (I). (B, D, F, H and J) (CC114‐78) Sulcal perivascular tau with equivalent and strong expression of AT8 (B) and CP13 (F) in neurones and threads. (D) AT180 was strongly expressed but in the neuronal cell bodies with less expression in threads and cell processes. AT100 and PHF1 both showed markedly lower neuronal expression with co‐localisation in some cells (arrows). Vessels in all cases are indicated by an asterisk and DAPI nuclear counterstain applied. Bar = 200 microns.

**FIGURE 4:**
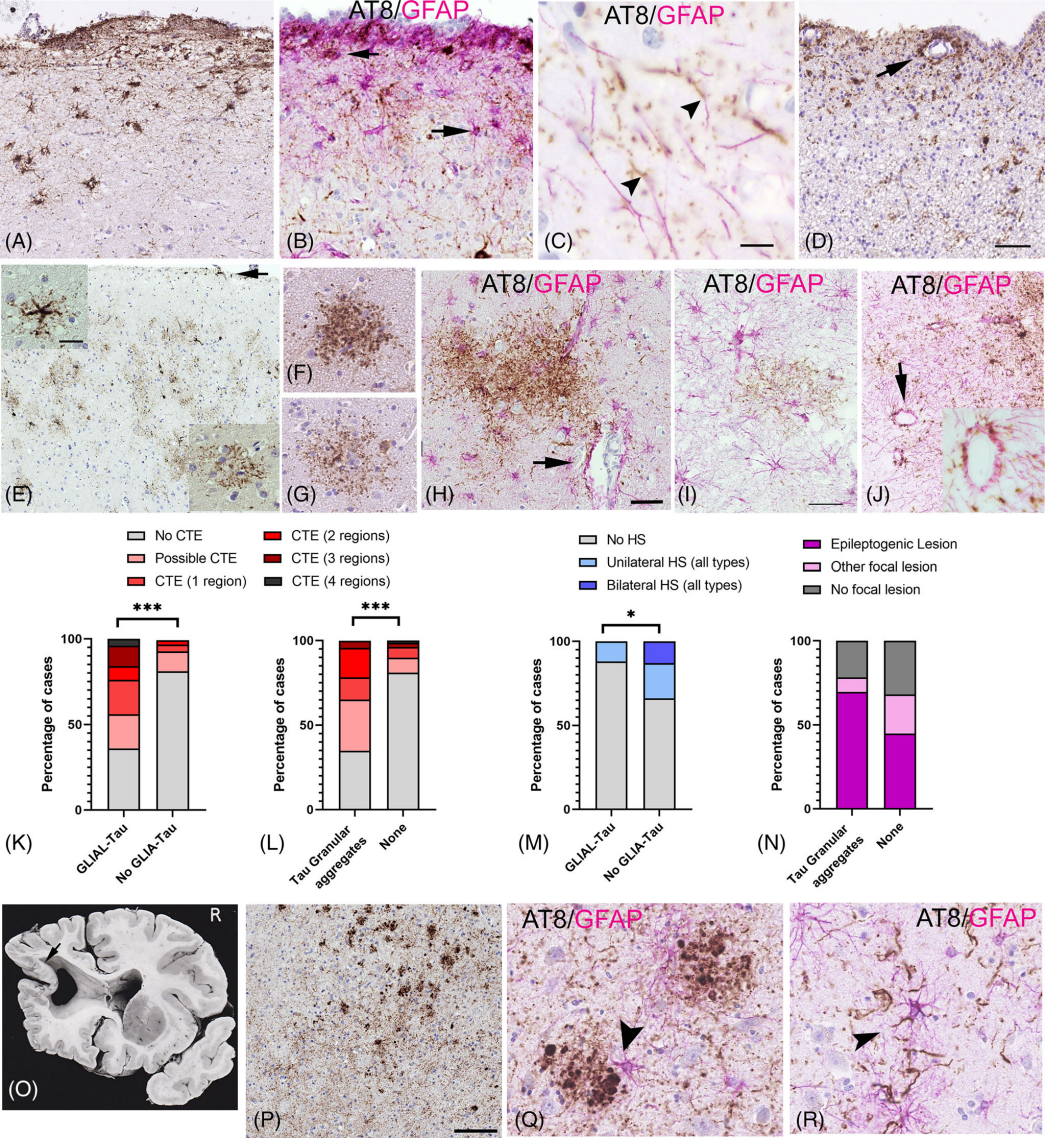
Astroglial tau pathology with AT8 immmunolabelling and GFAP/AT8 double labelling. (A) (CC44‐95) Subpial and layer I tau‐positive thorn‐shaped astrocytes with AT8 labelling from case summarised in Figure 2B with post‐traumatic epilepsy. (B) (CC23‐72) Subpial and marginal layer astrocytosis confirmed with GFAP with co‐localisation of AT8 in some cells (arrows) in a 65‐year‐old male with seizures following a head injury in childhood. (C) (CC 48‐87) AT8 showing beaded and intermittent labelling along radial subpial fibres in the superficial cortex from a 37‐year‐old male with CTE‐NC brain lesions (case summarised in Figure 2D). Bar = 10 microns. (D) (CC97‐76) Subependymal astroglia tau with AT8 with some aggregation in processes around vessels from a 46‐year‐old female with early onset of epilepsy, severe learning disability, Dandy–Walker malformation and hydrocephalus. Episodes of status epilepticus were reported but no head injury or CTE‐NC were noted in the cortex. Bar = 50 microns. (E) (CC22‐69). Superficial frontal cortex with subpial atroglial tau (arrow), scattered small neurones, glia and meshwork clusters shown in insets in a 79‐year‐old male with childhood onset seizures, neurodevelopmental delay, without a confirmed focal pathology or CTE‐NC features (Inset bar = 25 microns). (F, G) (CC97‐76, case as D) Granular aggregates of tau in the neuropil of varying density, without central core, but occasionally in proximity to positive neurones, glia or capillaries. (H) (CC23‐72, case as in B). Loose granular aggregates and meshworks in the amygdala, intermingled with GFAP astrocytes and AT8 processes extending around capillaries (Bar = 50 microns). (I) (CC61‐93) A 67‐year‐old with late‐onset epilepsy following an infarct with no definite CTE‐NC confirmed but mild periventricular tau in temporal lobe and a few loose granular clusters in the subiculum with proximity to intermingled GFAP positive cells and processes, although precise overlap not confirmed (Bar = 30 microns). (J) (CC121‐78) Sulcal glial and perivascular tau aggregates in a 63‐year‐old male with onset of epilepsy age 13 and resident at Chalfont epilepsy centre with unilateral hippocampal sclerosis confirmed and CTE‐NC; inset at higher magnification of perivascular tau. (K) Bar graph showing the percentage of cases with glial tau in relation to CTE‐NC which was significantly higher than those without astroglial tau pathology. (L) Bar graph of the percentage of cases with tau granular aggregates in relation to CTE‐NC which was significantly higher than those without this tau pattern. (M) Bar chart of the percentage of patients with glial tau in relation to hippocampal sclerosis (HS) pathology; in this group, less tau was noted than cases without HS. (N) Bar chart of tau granular aggregates in relation to focal pathology, with a trend for an increase in association with an epileptogenic pathology but this was not significant. (O, P) (CC127‐80). (O) Sixty‐eight male with longstanding epilepsy and resident at the Chalfont epilepsy centre with a complex brain malformation of porencephaly with polymicrogyria in the rim, a cystic lesion in the third ventricle and hydrocephalus. Globular aggregates of AT8 labelling and axons in the gliotic thalamus but no evidence of CTE‐NC in available blocks (Bar = 10 microns). (Q, R) (CC34‐96) Globular AT8 aggregates in the amygdala in proximity to GFAP‐positive astrocytes (arrow in Q) and in processes (arrow in R) in a 67‐year‐old male with onset of epilepsy at age 55 (termed temporal lobe epilepsy), memory decline and no focal pathology or CTE‐NC. ***p ≤0.001, *p <0.05 (Fisher's exact test); Graph N, *p* = 0.057.

We categorised 16 cases (15.7%) as CTE‐NC. A further, 14 cases (13.7%) showed suggestive features, as sulcal pTau, but did not meet definitive CTE criteria, and 72 cases had no CTE pathology (Figure 1). In all these three groups a mean of 3–4 blocks/case were available for evaluation. In CTE‐NC cases, 8/16 showed pathognomonic lesions (perivascular sulcal neuronal/glia tau) in one cortical region (Figure 2A), 1/16 in two cortical regions (Figure 2B), 3/16 in one cortical region and the hippocampus (Figure 2C) and 4/16 in two or more cortical regions and the hippocampus (Figure 2D). Of the six cases classed as PTE, two had CTE‐NC (Figure 2B); an absence of beta‐amyloid was noted in all PTE cases (Figure 1). pTau was present at old contusion sites, in neurones, subpial and parenchymal glia and threads, in seven cases with probable CTE‐NC and in one case without CTE‐NC (Figure 2E). Characterisation of tau phosphorylation sites in CTE‐NC cases, sulcal perivascular deposits, showed an equivalent distribution of CP13 to AT8; AT180 was present in cell bodies more than in neuropil threads whereas AT100 and PHF1 showed lower levels than AT8 with co‐expression in some positive neurones (Figure 3).

In relation to the epilepsy history, a younger age of onset of epilepsy was present in CTE‐NC compared to cases without (*p* = 0.039) (Figure 2G, Table 1). There was no relationship with the duration of epilepsy, but CTE‐NC cases had a younger age at death than cases without evidence of CTE‐NC (*p* = 0.008) (Figure 2G), and males were over‐represented (75% of cases) (*p* = 0.034). Of note, most of CTE‐NC cases were born between 1921 and 1940 than during any other time period compared to cases without. There was no observed relationship between CTE‐NC cases and seizure types or refractoriness. Memory impairment or cognitive decline was recorded in seven cases (43%), developmental delay in five (31.2%) and CTE‐NC cases were more often residents at an epilepsy care home (81.3%) compared to cases without CTE‐NC (54%). In CTE‐NC cases, a prior history of head injury was reported in 50% and evidence of TBI on neuropathology examination (62%) was increased eightfold compared to no CTE cases (16%, *p* = 0.0004). Of note, 16.7% of cases with documented TBI, including histories including multiple events, showed no pTau abnormalities. One patient with CTE‐NC (Figure 2C) was noted to have won trophies for boxing but detailed information on sports participation was not recorded for other cases. Another patient who sustained a penetrating brain injury during WW1 showed no CTE‐NC, although only one cortical region was available for evaluation (Figure 2F). Possible SUDEP as a cause of death was also more frequent in the CTE‐NC group (37%) than without CTE (14%) (*p* = 0.036) (Figure 2G). Following multivariate analysis, only the pathology‐confirmed TBI and institutional residence was significant between the CTE‐NC and no CTE groups (Table 1).

**TABLE 1:** Comparison of clinical, pathological and tissue factors between epilepsy cases with chronic traumatic encephalopathy‐neuropathology change (CTE‐NC) and without using logistic regression analysis.

	Observation	No CTE *n* = 72	No. cases data	CTE‐NC *n* = 16	No. cases data	OR	95% CI	Sig.
Clinical factors	Age onset (yrs)	32.59 (25.1)	58	15.1 (14.8)	12	0.96	0.924–0.998	*p* = 0.039
	Age death (yrs)	63.8 (13.9)	72	52 (17.5)	16	0.949	0.913–0.986	*p* = 0.008
	Duration (yrs)	26.4 (22.9)	50	31.8 (11.23)	10	1.012	0.98–1.04	*p* = 0.468
	Sex male	44%	72	75%	16	3.75	1.1–12.74	*p* = 0.034
	History of ‘Dementia’	30.60%	72	46.70%	16	1.98	0.64–6.1	*p* = 0.23
	Developmental delay	15.30%	72	31%	16	1.84	0.5–6.7	*p* = 0.35
	Institutional residence	54.20%	72	81.30%	16	3.36	0.96–13.97	*p* = 0.057*
	History of head injury	12.50%	72	43.80%	16	5.4	1.62–18.25	*p* = 0.006
	TBI (pathology confirmation)	16.70%	72	62.50%	16	8.3	2.54–27.3	*p* = 0.0004*
	SUDEP	14%	72	37.50%	16	3.6	1.0–12.3	*p* = 0.036
Pathology findings	Epileptogenic brain lesion	46%	72	62.50%	16	1.9	0.63–5.84	*p* = 0.25
	HS (all types)	29.20%	72	37.50%	16	1.4	0.47–4.52	*p* = 0.51
	Glial tau identified	12.50%	72	68.80%	16	15.4	4.3–54.6	*p* = 0.000013
	Granular aggregates	11.10%	72	50%	16	8.0	2.35–27.23	*p* = 0.0008
Tissue factors	PMI (days)	1.8 (1.2)	56	2.4 (1.8)	12			*p* = 0.288~
	Fixation time (days)	131.7 (204)	31	105.7 (62.5)	8			*p* = 0.265~
	Time in FFPE (years)	42.6 (7.8)	72	40.3 (7.5)	16			*p* = 0.395~

*Note*: *Only these clinical factors were significant following multivariate analysis of the seven significant clinical factors on univariate analysis. ~Mann–Whitney test.

Abbreviations: CI, confidence interval; FFPE, original formalin‐fixed paraffin‐embedded; HS, hippocampal sclerosis; PMI, post‐mortem interval; OR, odds ratio; SUDEP, sudden and unexpected death in epilepsy; TBI, traumatic brain injury.

#### 3.4.2 Astroglial tau patterns

Twenty‐five cases (24.5%) had focal astroglial tau pathology, including subpial (15 cases) (Figure 4A–C), periventricular (15 cases; 7 cases also showing subpial labelling) (Figure 4D) and in the amygdala region (Figure 4H,Q,R), with an age range of 37–89 (mean 61 years). pTau of any type was present in the amygdala in 75% of cases, where the tissue was available. AT8/GFAP double labelling confirmed focal tau labelling in astroglia cell bodies including thorn‐shaped astrocytes (Figure 4B), subpial process (Figure 4C) and perivascular end‐feet (Figure 4H,J). Other tau phosphorylation sites confirmed expression of AT100, AT180, CP13 and PHF1 in the subpial layer and layer I astrocytes but with a greater abundance of CP13 and AT100 (Figure 3A,C,E,G,I). Logistic regression analysis showed more glial tau pathology in males than females (*p =* 0.02, Table S3) but not associated with age at death. Glial tau was more frequently seen in CTE‐NC cases (68%) than no CTE (12%) (Figure 4K, Table 1, *p* = 0.00001) and less frequent in HS than non‐HS cases (*p* = 0.037, Fisher's exact test, Figure 4M).

#### 3.4.3 Tau granular cortical aggregates

Twenty‐three cases (22.5%) showed localised, fine tau granular aggregates or meshworks in the neuropil that were not clearly associated with a cell body (neuronal or glial) or amyloid core. These were noted in isolation (Figure 4F,G,I) or in clusters, including a preference for the grey matter and superficial cortical layers (Figure 4E), in proximity to capillaries (Figure 4H) but occasionally noted in white matter. Double labelling with GFAP confirmed proximity with glial cells, processes and perivascular end‐feet but no clear co‐localisation (Figure 4H,I). Tau‐positive granular aggregates were associated with male gender but not age (*p* = 0.01, Table S3) and with CTE‐NC (*p* = 0.001, Figure 4L, Table 1); there was a trend for an association with an identified epileptogenic brain lesion (*p* = 0.057, Fisher's Exact test, Figure 4N). Three cases showed an unusual pattern and distribution of non‐neuronal, glial‐like and globular tau which was not further characterised due to limited regions available (Figure 4O–R).

#### 3.4.4 Alzheimer's disease neuropathology changes

**FIGURE 5:**
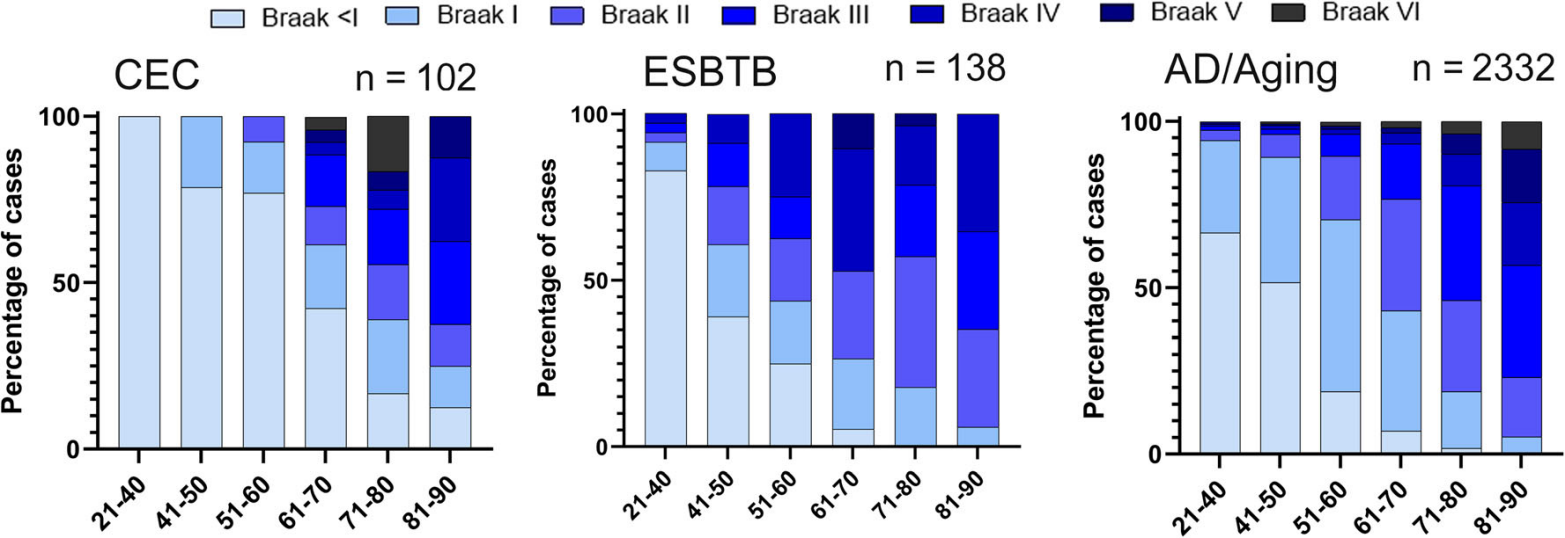
Braak staging in the Corsellis Epilepsy Collection (CEC) across age groups. The staging in the current cohort was compared to (i) a large published unselected autopsy series of ageing and Alzheimer's disease (AD) shown across age decades; in this series, there was no documentation of any seizures or epilepsy in 2332 cases [23] and (ii) our previously published study in a cohort of 138 epilepsy brains with mixed underlying aetiologies from the epilepsy brain and tissue bank (ESBTB) at UCL [9]. In younger adults (21–40 years), high Braak stages (III‐VI) were all low in all series, at less than 6%. In the 51–60 age group an increased representation of high Braak stages in the ESBTB epilepsy cohort (37.5%) compared to AD/ageing non‐epilepsy cohort (9.9%) was not observed in the CEC cohort. In the 61–70 age group, the percentage of cases with high Braak stages was higher in the CEC (45%) than AD/ageing (42%) but not as high as the ESBTB series (47%). In the older age groups (71–90), the representation of high Braak stages was lower in both epilepsy cohorts (62%–64%) than AD/ageing (85%).

Braak staging in the CEC cohort, based on the AT8 labelling in the hippocampus and available cortical regions, was estimated in 84 cases and compared to data from a large unselected non‐epilepsy ageing cohort which included AD cases (*n* = 2332) [23] as well as to our previous epilepsy post‐mortem cohort [9] (Figure 5). The CEC cohort had a higher percentage of cases in the young to mid‐adulthood range (21–60 years) with lower Braak stages (Stage 0‐II) than the non‐epilepsy cohort (Mann–Whitney test, *p* <0.05). There was a trend for increased high Braak stages (Stages III‐VI) in mid‐late adulthood (61–70 years) but a reduced number of high Braak stages for the oldest in epilepsy (71–90 years) compared to non‐epilepsy series.

LOE was associated with higher Braak stages (*p* <0.01, OR 2.0, 95% CI 1.1–3.3) and a documented history of dementia (*p* <0.001, OR 4.9, 95% CI 1.9–12.6) (Figure 1). Beta‐amyloid staining carried out in 64 cases of the series revealed Aβ‐related pathology in 30 with diffuse and/or core plaques and additional amyloid angiopathy in five in keeping with AD neuropathology change (AD‐NC). Seven cases without beta‐amyloid accumulation or astroglial tau and with tau limited to the mesial temporal lobe were considered to represent primary age‐related tauopathy (PART; mean age 66.5 years, range 42–88) with a mean age of onset of epilepsy of 25 years (range 4–65). Of note, only 14.3% of suspected PART cases had LOE compared to 100% of cases with Braak stage VI.

#### 3.4.5 Epilepsy‐specific patterns of tau

**FIGURE 6:**
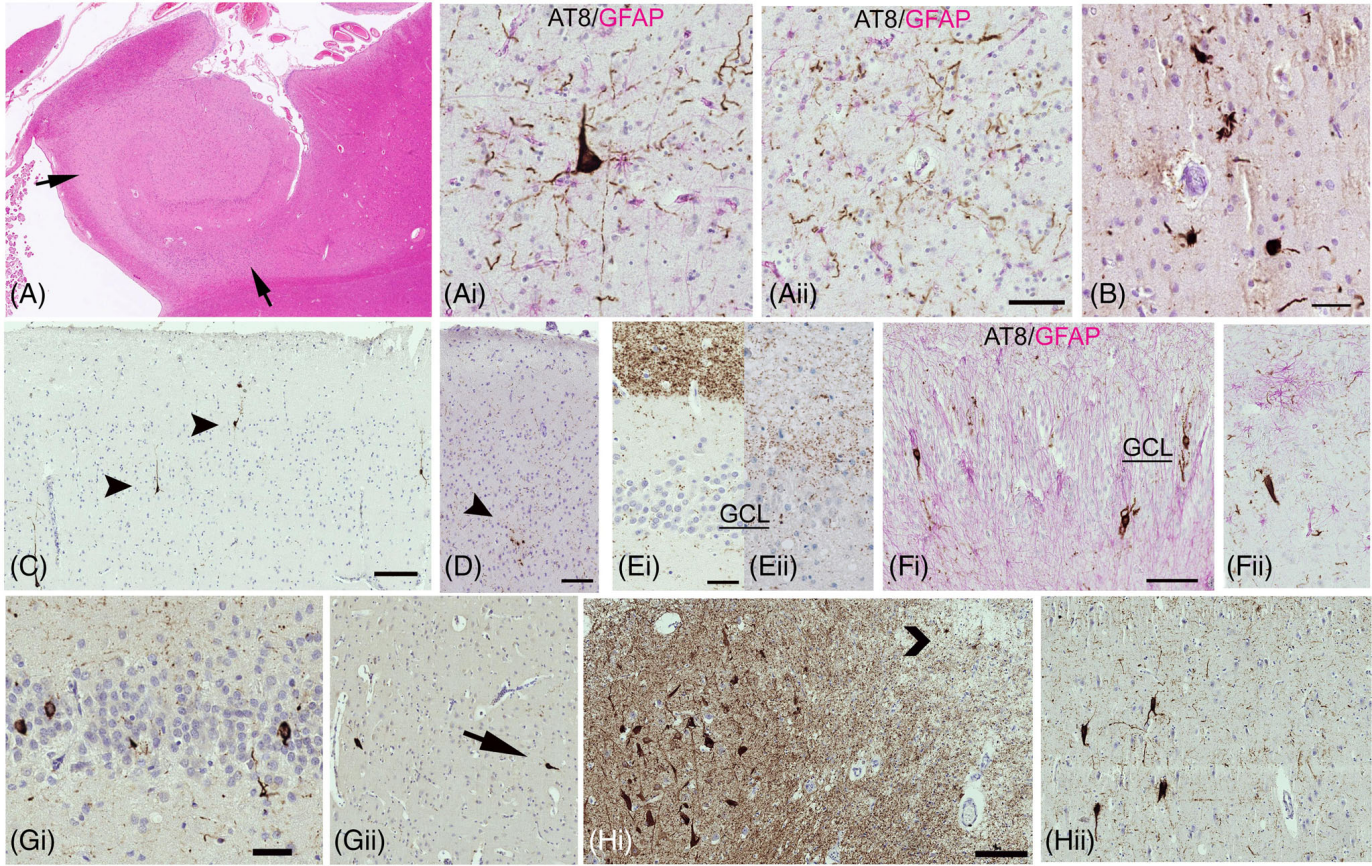
Non‐Braak and non‐CTE AT8 distribution in epilepsy cases. (A) (CC95‐81) 61‐year‐old male with age of onset of seizures age 5, following meningitis, with bilateral hippocampal sclerosis (HS) (shown as H&E on one side in A with neuronal loss in CA1 [arrows] and CA4 and subiculum sparing), resident at an epilepsy home with 30 complex partial seizures/year. There were overall low tau loads on AT8 in the hippocampus but equivalent labelling in the inferior temporal gyrus (Aii) to the parahippocampal gyrus (Ai) but without a sulcal or perivascular predilection. Bar = 110 microns (B) (CC103‐78). A 55‐year‐old male with onset of seizures age 11, with 2003 seizures reported/month and resident in an epilepsy care home. He developed a dementia syndrome later in life, and small vessel disease was noted at post‐mortem, microinfarcts with minimal tau in mesial temporal lobe but scatted tau in the superficial inferior temporal gyrus but not in the typical distribution for CTE‐NC. Bar = 50 microns. (C) (CC37‐68) A 45‐year‐old male with childhood onset of epilepsy and bilateral asymmetrical HS and developmental delay. There was asymmetry in the cortical tau which was present in superficial cortical neurones in the temporal neocortex, with a focal sulcal pattern, on the side with HS type 1 but not on the side with HS type 2. There was minimal mesial temporal AT8 labelling. Bar = 200 microns. (D) (CC86‐79). A 46‐year‐old male with a brain malformation and onset of epilepsy at 26 years with AT8 labelling in the fusiform gryus in superficial layer neurones and threads equivalent to labelling in subiculum and parahippocampal gyrus. Bar = 100 microns. (E) (CC121‐78) A 72‐year‐old female with onset of seizure at age 2 with unilateral HS and an old infarct. (Ei) In the dentate gyrus on the non‐sclerotic hippocampus, AT8 was greater in the outer than inner molecular layer, whereas Bar = 30 microns (Eii) on the side with Type 1 HS there was diffuse axonal labelling in the molecular layer in keeping with a pattern of mossy fibre axonal reorganisation. (F) (CC33‐67) An 83‐year‐old female with late onset of epilepsy at 68, with generalised seizures and unilateral Type 1 HS (AT8/GFAP); AT8 labelling was not overall high but scattered neurones throughout the hippocampal subfields and dentate gyrus granule cells shown between the radial glial fibres (Fi), were greater than in the subiculum (Fii) and entorhinal cortex. Bar = 100 microns. (G) (CC40‐80). An 89‐year‐old male with lifelong epilepsy and runs of seizure documented up to death and resident at the Chalfont centre; there was a CTE‐NC pattern in some regions and the hippocampus, which was not sclerotic, had a relatively greater tau load than the parahippocampal gyrus, including the granule cells (Gi). A region of polymicrogyria was noted in the insular cortex which also showed occasional scattered AT8‐positive neurones (Gii) in addition to subpial glial in this region. Bar = 50 microns. (Hi, Hii) (CC44‐95) Difference in AT8 between mesial temporal blocks shown in CA1 with greater labelling on the side with scar (chevron). GCL, granule cell layer. Bar = 200 microns.

The distribution of tau in some cases was not typical for Braak stage progression [24] or CTE patterns [13] and may represent epilepsy‐specific accumulation of pTau, related to neuronal activity or altered networks. In four cases, AT8 labelling in superficial neocortical neurones on the gyral crown was greater than in the temporal allocortical regions (Figure 6A–D). In four cases with HS, AT8 labelling in the dentate molecular layer was in keeping with sprouted mossy fibres (Figure 6E) as previously described [6, 9]. Higher tau burden in granule cells relative to other hippocampal subfields (Figure 6F,G), AT8 associated with cortical brain lesions and asymmetrical level of tau between hemispheres, may be related to seizure activity (Figure 6H); however, precise information of seizure lateralisation or onset was not available to further explore this.

Of note, nine epilepsy cases with no AT8 labelling included cases with long duration (>40 years) of epilepsy, poor seizure control and evidence of prior TBI. When compared to cases with tau, the cases with no pTau were, however, significantly younger at the time of death (*p* <0.05). Post‐mortem interval or fixation times were not significantly longer in cases with no pTau labelling but we noted significantly longer ages of the processed tissue samples (Table S4).

## 4 DISCUSSION

In this historical post‐mortem epilepsy archive, we observed a prevalence of CTE‐NC of 15.7%, which was associated with TBI. In addition, higher Braak stages were associated with late‐onset epilepsy and memory decline, although not more frequent than in a large non‐epilepsy ageing/AD cohort. We also noted a group with a long epilepsy history and TBI with no evidence of tau pathology, suggesting tau accumulation is not inevitable.

### 4.1 CTE‐NC prevalence in epilepsy compared to other cohorts and sports injury

Addressing the prevalence and patterns of tau pathology in epilepsy post‐mortem series has advantages over surgical series, as both the causes and severity of epilepsy are more varied, which can shed light on factors that could promote neurodegeneration. The Corsellis tissue collection enabled the discovery of the pathology now termed CTE [25], and a contemporary analysis of tau pathology in these cases was recently published [18]. Our study focuses on patients with epilepsy in the Corsellis collection, identifying CTE‐NC in 15.7%. In a further subset of 13.7%, we noted features of CTE which did not meet minimal diagnostic criteria [13]; similar cases reported in other series are argued to represent early CTE‐NC stages [26]. CTE‐NC in 15.7% of epilepsy cases is higher than reported in community‐based autopsy series (0%–8%, median 1.75%) and neurodegenerative/dementia brain banks (0.79%–11.9%, median 5.8%), but less than in series of contact sports participants (32%–87%, median 67%) (Table 2). In our previously published epilepsy post‐mortem cohort of mixed aetiologies (from the Epilepsy Brain and Tissue Bank at UCL), which predated recent diagnostic criteria for CTE, we noted astroglial tau in 35%, sulcal tau in 5.8%, perivascular tau in 6.5% and a correlation of tau load with a history of TBI [9]. In a routine neuropathology practice, CTE‐NC was recently identified in 4/180 cases, two of whom had refractory epilepsy [14] and a single case of PTE with CTE was reported in the original Boston TBI series by McKee et al. [12] (Table 2). In surgical lobectomies from patients with refractory epilepsy, ‘CTE‐like’ patterns have occasionally been reported [15, 16, 39] all suggesting that refractory seizures can be a risk factor for CTE. Based on available information, we observed no direct relationship of CTE‐NC with seizure type and severity in the Corsellis Collection; although there was an association of CTE‐NC with younger age of onset, institutionalisation for epilepsy and SUDEP, all of which may suggest more poorly controlled epilepsy, these factors could also equally implicate a greater susceptibility to head injury.

**TABLE 2:** Reports of prevalence of neuropathological chronic traumatic encephalopathy (CTE) in 17 selected post‐mortem series.

Reference	Year	Cases	Source of material	Population selected	Epilepsy	Mean age (range in years)	CTE criteria applied	% CTE	% ARTAG	% Beta‐amyloid pathology
Mez et al. [27]	2017	202	VA‐BU‐CLF Brain Bank Boston University	American football players (all levels)	No reported cases	67 (52–77)	1st NINDS‐NIBIB consensus criteria for CTE (2016)	87%	% not specified	91% of stage IV CTE (DP > NP, CAA)
McKee et al. [12]	2013	85	VA‐BU‐CLF Brain Bank Boston University	Repetitive mild TBI (80 athletes)	1% (PTE)	54.1 (14–98)	Based on previous studies of CTE	80%	Not stated	27.4% of CTE cases
Goldfinger et al. [18]	2018	14	Corsellis Brain Collection, UK	Boxers (from study [17])	No reported cases	68.4 (17–95)	2nd NINDS‐NIBIB consensus criteria for CTE (2016)	50%	71.4% (100% of CTE cases)	Not stated
Ling et al. [28]	2017	6	Queen Square Brain Bank, UCL, London	Soccer players with dementia	GS reported in 1 case, advanced dementia but no CTE	72 (65–83)	1st NINDS‐NIBIB consensus criteria for CTE (2016)	67%	100%	100%
Schwab et al. [29]	2021	35	Canadian Football Alumni Association	Hockey and Football players	No reported cases	59.9 (CTE), 65 (No CTE)	1st NINDS‐NIBIB consensus criteria for CTE (2016)	49%	100% in CTE cases	AD‐NC 62.5%
Stewart et al. [30]	2023	31	UNITE Brain Bank, Boston University US, Glasgow TBI Sports Brain Bank UK, Australian Sports Brain Bank, Royal Prince Alfred Hospital, Australia	Rugby Union players	No reported cases	60.4 (17–95)	2nd NINDS/NIBIB consensus criteria for CTE, 2021	68%	Not stated	Not stated
Bieniek et al. [31]	2015	66	Mayo Clinic Neurodegenerative brain bank, US	History of Contact sport	No reported cases	73 (41–93)	2013 McKee stages (I‐IV)	32%	% not specified	Not stated
Ling et al. [32]	2015	268	Queen Square Brain Bank, UCL, London, UK	Consecutive neurodegenerative cases (inc. 47 controls), 93% of CTE TBI history	No reported cases	81 (61–95) with CTE	2013 McKee stages (I‐IV)	11.9%	Not stated	72%
Walt et al. [33]	2018	155	Veterans Affairs Biorepository Brain Bank, USA	ALS	No reported cases	70.3	1st NINDS‐NIBIB consensus criteria for CTE (2016)	5.8%	44% (of CTE cases)	55% (of CTE cases)
McCann et al. [26]	2022	636	Sydney Brain Bank, Australia	Focus on dementia and ageing (TBI history in 109)	No reported cases	79 (74–92) with CTE	1st and 2nd NINDS/NIBIB consensus criteria for CTE	0.79%	100% (with CTE)	100%
Postupna et al. [34]	2021	532	Adult changes in thought (ACT) study, Washington, US	Community‐based study, 107 with history of TBI + LOC	No reported cases	86.9 (TBI cohort);87.9 (No TBI group)	1st NINDS‐NIBIB consensus criteria for CTE (2016)	0.6% (none with TBI)	5%	% not specified
Bieniek et al. [35]	2020	750	Mayo Clinic Tissue Registry, US	300/750 sports participants	No reported cases	73 (33–91) with CTE	1st NINDS‐NIBIB consensus criteria for CTE (2016)	3%	Not stated	Not stated
Adams et al. [36]	2018	164	Framingham Heart Study Cohort, US	Community‐based ageing cohort, (11.6% contact sports)	No reported cases	87.2 (57–105)	1st NINDS‐NIBIB consensus criteria for CTE (2016)	1%	Not stated	Not stated
Forrest et al. [37]	2019	310	Vienna Trans‐Danube Ageing (VITA) study, Austria	Ageing cohort, 8% history TBI	No reported cases	83 (76–91)	1st NINDS‐NIBIB consensus criteria for CTE (2016)	0%	38%	Not stated
Noy et al. [38]	2016	111	Community‐based neuropathology referrals, <60 years, Winnipeg, Canada	44% history of TBI	1 case with CTE, Late‐onset epilepsy (SUDEP?), Also AD‐NC	51.7 (42–84) CTE group	1st NINDS‐NIBIB consensus criteria for CTE (2016)	8% (stage I+)	Not stated	11%
Suter et al. [14]	2022	180	Neuropathology Dept, Royal Prince Alfred Hospital, Sydney, Australia	Routine referrals: 18 dementia, 26 histories of TBI	2 CTE cases, refractory epilepsy	55 (18–101)	2nd NINDS/NIBIB consensus criteria for CTE, 2021	2.5%	25% (of CTE cases)	Not stated
Current CEC series		102	ERUK, Corsellis Epilepsy collection, UCL, UK	History of epilepsy, varying severity/cause, 22.5% TBI	100% of cases	52 (25–89) CTE group	Based on 2nd NINDS/NIBIB consensus criteria for CTE, 2021	15.7%	24.5% (subpial, periventricular and amygdala)	54% CTE 72% No CTE

*Note*: Highlighted in grey as contact sports players, white in neurodegenerative disease brain banks and darker grey in community post‐mortem series (current series in blue). This is not a comprehensive inclusion of all published studies. The criteria used for CTE diagnosis include Mckee stages of CTE 2013, 1st NINDS‐NIBIB consensus criteria for CTE and 2nd NINDS/NIBIB consensus criteria for CTE.

Abbreviations: ALS, amyotrophic lateral sclerosis; CAA, cerebral amyloid angiopathy; CTE, chronic traumatic encephalopathy; DP, diffuse plaques; ERUK, Epilepsy Research UK; GS, generalised seizures; LOC, loss of consciousness; NP, neuritic amyloid plaques; TBI, traumatic brain injury; VA‐BU‐CLF, Unite brain collection based at Boston University.

We observed a relationship of CTE‐NC with a history of head injury and neuropathological confirmation of old TBI. As only six patients in our cohort had a suspected PTE diagnosis (50% of these with CTE‐NC), most brain injuries are likely sequalae of seizures, as noted in our previous cohort [9]. We also observed pTau at contusion sites which contrasts with an absence of tau in contusions in a previous non‐epilepsy series of CTE [38]. We did not have details of contact sports participation, but one CTE‐NC case (Figure 1C) had a history of boxing in addition to epilepsy. Detailed neuropsychological and cognitive information was also not available for further characterisation of ‘traumatic encephalopathy syndrome’ [40]. Importantly, it has been argued that sports injury brain banks have a selection bias for case recruitment and are not population based, thus overestimate the true prevalence of CTE in the community [26]. Similarly, the Corsellis collection, as well as our previous post‐mortem study [9], likely over‐represent more severe and refractory epilepsy and the incidence of 15.7% with CTE‐NC cannot be extrapolated to the general population with epilepsy, particularly in the modern era with the advent of better seizure management, new ASMs, surgical treatments and neuroprotective strategies. Nevertheless, as a third of adult patients with epilepsy remain refractory to currently available ASMs [41] our findings remain relevant to modern clinical management.

### 4.2 Tau, Alzheimer's disease and questions on epileptogenesis

Even though the cellular mechanisms promoting epileptogenesis are not fully understood, tau has been implicated in this process [1, 42, 43], and furthermore, a bidirectional relationship between AD and LOE is recognised [7]. We did not observe an increase in Braak stage in the Corsellis Epilepsy collection compared to a large ageing cohort (which included dementia/AD cases) but we noted an association with LOE whereas cases categorised as PART were not associated with LOE. In PART, pTau is restricted to mesial temporal lobe structures with an absence of beta‐amyloid, the cognitive change is mild, and unlike AD‐NC, a clear link with epilepsy has not been reported [44, 45]. It is also noteworthy from the literature that seizures are only infrequently reported in CTE syndrome (Table 2). In a study of 14 soccer players with dementia, one had seizures at an advanced disease stage (AD‐NC Braak stage IV) but CTE pathology was not confirmed [28]. In the original study of the ‘aftermath of boxing’, with detailed neurological histories of 15 cases, none had epilepsy or seizures [17], nor is it documented in reviews of recent large CTE series [25, 46] or part of traumatic encephalopathy syndrome [40, 47]. We propose that a history of seizures, likely through its increased risk of head injury, should be included in clinical assessments for CTE risk factors in addition to sports participation. However, these observations may also imply a more unidirectional relationship, that is, that epilepsy can increase the risk of CTE, but not necessarily vice versa. This prompts further questions on how pathological tau is considered pro‐epileptogenic in AD but less so in CTE. This may related to differences in pTau cellular distribution [48], its conformation [49] or post‐translational modification [50]. In a recent study of CTE, higher pTau202/396 ratios were noted in CTE compared to AD [50] and we similarly observed greater neuronal labelling for CP13 and AT8 (pTau202) than PHF1 (pTau 396), particularly in perivascular neurones, warranting further investigations.

### 4.3 Astroglial tau in epilepsy; relationship to CTE and ARTAG

Ageing‐related tau astrogliopathy (ARTAG) pathological tau accumulation is restricted to astroglia and comprised of 4R isoforms, but without a clearly defined cognitive syndrome [51, 52]. We noted tau astrogliopathy in 24% of our epilepsy cases; we were unable to determine 3R/4R isoform composition but noted no relationship with age but with CTE‐NC. Granular aggregates of tau seen mainly in the grey matter in 22.5% in epilepsy cases, resembled ‘granular fuzzy’ astrocytes of ARTAG [52] but did not localise to astroglia on double labelling. They also bore resemblance to ‘dense dot like aggregates’ of tau described in CTE associated with blood vessels [10]. Tau granular aggregates in epilepsy were also significantly associated with CTE‐NC but not age, epileptogenic lesion or cognitive decline. Although not part of the diagnostic criteria of CTE [13], there is a close association between ARTAG and CTE [53] and they may represent a spectrum of the same disorder with ARTAG variably reported in 25%–100% of CTE cases [14, 18, 29, 32, 33] (Table 2). We also noted glial tau in 35% in a previous epilepsy post‐mortem study [9], non‐glial cortical tau granular aggregates in 15% in a TLE surgical series [6], and in the current series, the later were also noted with epileptogenic lesions warranting further characterisation of their relationship and cellular origin in both traumatic lesions and epilepsy.

### 4.4 Atypical pTau patterns in epilepsy

Atypical (non‐Braak/non‐CTE) tau patterns noted in this epilepsy series included involvement of superficial gyral neurones, asymmetrical patterns, preferential neocortical involvement over mesial temporal regions and hippocampal mossy fibres which may all reflect epileptogenic activity. Tau phosphorylation is tightly regulated by NMDA receptor activation [54] with tau phosphorylation following seizures in experimental models [55]. pTau in superficial cortical neurones has been noted in surgical epilepsy lobectomies [6, 16, 56] and may reflect epileptogenic networks. This distribution also lends comparisons to the superficial gyral cortical tau accumulation described in Nodding syndrome, a severe endemic form of atonic epilepsy [57] and subacute sclerosing panencephalitis, in which cryo‐EM showed an identical tau filament structure to CTE [58]. As yet, there is no evidence of any distinct tau conformations in epilepsy. Importantly, we identified a group of patients with no AT8 labelling with similar clinical histories to those with tau, apart from a slightly younger age. Twenty per cent of McKee's original 2013 study of 86 cases with history of repetitive TBI did not have CTE [12] which could imply neuroprotective factors in a subset of patients.

### 4.5 Study limitations

There are several limitations to this study from working with a historical tissue collection, primarily the lack of complete data on cases regarding epilepsy histories and modern investigations for accurate disease classification of epilepsy syndrome. We used the original paraffin‐embedded tissue samples which were several decades old. We did not observe differences in Tau staining for fixation time (Figure S1) or post‐mortem interval but we cannot exclude effects of the age of the tissue samples influencing tau immunohistochemistry. We noted in cases which were negative for pTau that the paraffin blocks were older, and we cannot exclude that loss of antigenicity is a contributing factor in these cases. We were unable to stage CTE on NINDS/NIBIB criteria due to limited blocks available or to conduct more extensive analysis on all recommended regions [13]. Our regional sampling strategy is however comparable to CTE‐NC screening conducted in recent post‐mortem series [25, 35, 37]; nevertheless, we cannot exclude under‐detection of CTE‐NC as the pathology can be patchy. Finally, we focused on Braak staging and CTE‐NC pathology and did not further investigate or classify other tau/proteinopathies and neurodegenerative diseases.

## 5 CONCLUSIONS

This historical post‐mortem epilepsy series supports the hypothesis that different patterns of tau are noted and importantly recognises that a proportion, with similarly severe epilepsy have no tau pathology. TBI associated with seizures is a risk factor for CTE‐NC in some patients. This study underscores the further work needed on the genetic and environmental risk factors for tau accumulation in epilepsy and its clinical significance in contribution to any memory and cognitive decline.

## AUTHOR CONTRIBUTIONS

MR and AM conducted the immunohistochemistry and analysis. HEH restored the post‐mortem archive, sample preparation and clinical records of the brain collection. MR, AM, HEH, JL and MT carried out analysis of sections and related data and interpretation. MT and JL conceived the study design. All authors were involved in manuscript preparation.

## CONFLICT OF INTEREST STATEMENT

The authors declare that they have no competing or conflicting interests.

## Supporting information


**Figure S1.** AT8 immunohistochemistry across a range of fixation times (shown in days). Top two images are AT8 combined with GFAP.

**Table S1.** Case details of epilepsy history, pathology, stains conducted and post‐mortem data.

**Table S2.** Summary of likely underlying aetiologies for epilepsy following evaluation of clinical and pathology records. In some cases, more than one pathology was present, including malformations with more than one malformation type. In 51 cases, a focal, presumed epileptogenic pathology was identified. In 20 cases, a focal pathology was assumed as a secondary and acquired pathology based on time course of epilepsy.

**Table S3.** Multivariate logistic regression analysis for glial tau pathology in relation to clinical factors with significant factors shown in bold red and trends in red. Exp(B) = Odds ratio and 95% confidence interval (CI) shown.

**Table S4.** Comparison of clinical and tissue protocol variables in epilepsy cases with negligible pTau present to cases with pTau. Logistic regression analysis statistics shown for variables and 95% confidence intervals (CI), odds ratio and significant values in red.

**Data S1.** Supporting information.

## Data Availability

The data that supports the findings of this study are available in the supplementary material of this article.
