# Supplementary figures and images for: The prevalence of chronic traumatic encephalopathy in a historical epilepsy post‐mortem collection
Source: Brain Pathol. 2024 Nov 11;35(3):e13317. doi: 10.1111/bpa.13317 (PMC11961211; doi:10.1111/bpa.13317)

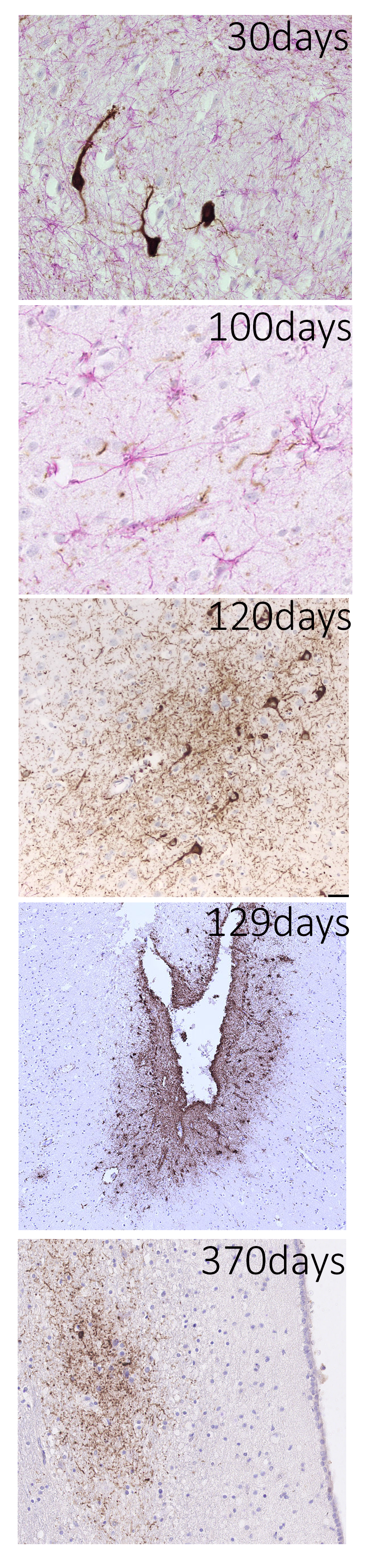

Supplement: Supplementary file 1 — Figure S1. AT8 immunohistochemistry across a range of fixation times (shown in days). Top two images are AT8 combined with GFAP. [file BPA-35-e13317-s006.tif]
