# Supplementary material for: The prevalence of chronic traumatic encephalopathy in a historical epilepsy post‐mortem collection
Source: Brain Pathol. 2024 Nov 11;35(3):e13317. doi: 10.1111/bpa.13317 (PMC11961211; doi:10.1111/bpa.13317)
Supplement: Supplementary file 2 — Table S1. Case details of epilepsy history, pathology, stains conducted and post‐mortem data. [file BPA-35-e13317-s001.docx]

| **CEC CASE NUMBER** | **YEAR OF PM** | **YEAR OF BIRTH** | **AGE AT DEATH** | **SEX** | **GENERALISED SEIZURES** | **FOCAL SEIZURES** | **AGE ONSET (YEARS), GROUPS** | **DURATION EPILEPSY (YEARS). GROUPS** | **FREQUENCY/CONTROL** | **SEIZURE MEDICATIONS** | **LIKELY AETIOLOGY OF EPILEPSY** | **HISTORY OF DEMENTIA, COGNITIVE, MEMORY DECLINE** | **RESIDENT AT INSTITUTE OR EPILEPSY COLONY** | **FALL/I INJURIES/ FRACTURES** | **TBI PATHOLOGY** | **DEVELOPMENTAL/LEARNING DELAY** | **FIXED BRAIN WEIGHT (IF N/A FRESH) Grams** | **HS DIAGNOSIS** | **MCD** | **CVD DIAGNOSIS** | **NUMBER OF BLOCKS EVALUATED WITH AT8** | **HIPPOCAMPAL BLOCKS** | **AMYGDALA** | **AMYLOID BETA** | **GFAP +AT8 DOUBLE LABELLING** | **PMI (DAYS)** | **FIX TIME (DAYS)** | **FFPE TIME (YEARS)** | **POSSIBLE SUDEP** |
| --- | --- | --- | --- | --- | --- | --- | --- | --- | --- | --- | --- | --- | --- | --- | --- | --- | --- | --- | --- | --- | --- | --- | --- | --- | --- | --- | --- | --- | --- |
| CC104-65 | 1965 | 1887 | 82 | M | Y | Y | >40 | 2to10 |  | NO DETAIL | ND/AD | Y | N | INJ | N | N | 1390 | NO HS | N | NO | 3 | 1 | 0 | Y | N |  | 46 | 57 | NON-ERD |
| CC169-66 | 1966 | 1940 | 26 | F | Y | Y | <10 | 10+ | PC | PHENOBARB. | PI | N | Y | NO | N | Y | 1002 | NO HS | N | PI | 2 | 0 | 0 | N | N | 1 | 32 | 56 | ERD |
| CC191-66 | 1966 | 1923 | 43 | M | Y | Y | <20 | 10+ |  | NO DETAIL | UK | N | Y | NO | N | Y | 1300 | NO HS | N | NO | 4 | 2 | 0 | N |  | 2 | 30 | 57 | NON-ERD |
| CC27-67 | 1967 | 1915 | 52 | F | Y |  | <10 | 10+ | PC | PHENOBARB. | UK | N | Y | NO | N | Y | 1435 | NO HS | N | NO | 1 | 0 | 0 | Y | Y | NA |  |  | SUDEP (POSS) |
| CC33-67 | 1967 | 1884 | 83 | F | Y | Y | >40 | 10+ | MC | NO DETAIL | O/S | Y | N | INJ | N | N | 1180 | TYPE 1 | N | RI | 3 | 1 | 0 | Y | Y | NA | 30 | 55 | NON-ERD |
| CC98-67 | 1967 | 1897 | 70 | M |  |  | <10 | 10+ |  | NO DETAIL | UK | Y | Y | HI | Y | N | 1115 | NO HS | N | CI | 1 | 0 | 0 | N | N | 1 | 20 | 55 | NON-ERD |
| CC147-67 | 1967 | 1907 | 61 | F | Y |  | >40 | 2to10 | PC | PHENOBARB., PHENYTOIN | UK | N | N | NO | N | N | 1190 | NO HS | N | AI | 4 | 1 | 1 | Y | N | 1 | 35 | 55 | ERD |
| CC37-68 | 1968 | 1923 | 45 | M |  |  | <10 | 10+ | MC | PHENOBARB., PHENYTOIN | HS | Y | N | NO | N | Y | 1225 | BILAT/ASYM | N | CI | 2 | 2 | 0 | N | N | 2 | 26 | 54 | NON-ERD |
| CC146-68 | 1968 | 1895 | 73 | F | Y | Y |  | 10+ | PC | PHENOBARB. | UK | N | Y | HI | Y | Y | 1260 | NO HS | N | NO | 1 | 0 | 0 | N | N | 3 |  | 54 | NON-ERD |
| CC22-69 | 1969 | 1890 | 79 | M | Y | Y | <10 | 10+ |  |  | UK | N | Y | INJ | N | Y | 1505 | NO HS | N | NO | 5 | 0 | 1 | Y | N |  |  | 54 | NON-ERD |
| CC45-69 | 1969 | 1907 | 62 | F | Y | Y | <10 | 10+ | PC | PHENOBARB., PHENYTOIN | UK | Y | Y | INJ | N | Y | 1000 | NO HS | N | NO | 2 | 2 | 0 | N | N | 5 | 120 | 53 | NON-ERD |
| CC58-69 | 1969 | 1927 | 42 | F | Y | Y | <10 | 10+ | PC | NO DETAIL | HS | N | N | INJ | N | Y | 915 | TYPE 1 | N | AI | 2 | 0 | 0 | N | N | 2 | 90 | 51 | NON-ERD |
| CC165-69 | 1969 | 1925 | 46 | F | Y | Y | 20-40 | 10+ | PC | PHENOBARB., PHENYTOIN | UK | N | N | HI | Y | N | 1280 | NO HS | N | NO | 3 | 1 | 1 | Y | N | NA |  | 53 | ERD |
| CC153-70 | 1970 | 1906 | 64 | F |  |  | <10 | 10+ |  | NO DETAIL | UK | N | Y | NO | N | Y | 1430 | NO HS | N | NO | 3 | 1 | 1 | N | N | 2 | 61 | 52 | NON-ERD |
| CC191-70 | 1970 | 1906 | 64 | F | Y |  | <20 | 10+ | PC | PHENOBARB., PHENYTOIN | UK | Y | Y | INJ | N | N | 1170 | NO HS | N | NO | 4 | 1 | 1 | N | N | 2 |  | 52 | NON-ERD |
| CC29-71 | 1971 | 1926 | 43 | M | Y |  | 20-40 | 2to10 | WC | PHENOBARB., PHENYTOIN | CVD | N | N | INJ | N | N | 1480 | TYPE 1 | N | RI | 2 | 0 | 0 | N | N | 0 | 110 | 52 | SUDEP (POSS) |
| CC60-71 | 1971 | 1888 | 83 | F | Y |  | >40 | 0-2 | PC | NO DETAIL | ND/AD | Y | N | INJ | N | N | 1170 | NO HS | N | CI | 3 | 1 | 0 | Y | N | 2 |  | 51 | NON-ERD |
| CC61-71 | 1971 | 1928 | 46 | M | Y | Y | <20 | 10+ | MC | PHENYTOIN | UK | N | N | INJ | N | N | 1470 | BILAT/ASYM | N | AI | 1 | 1 | 0 | Y | N | 0 | 60 | 51 | ERD |
| CC94-71 | 1971 | 1919 | 52 | M | Y | Y | 20-40 | 10+ |  | NO DETAIL | HS | Y | N | INJ | N | N | 1440 | TYPE 1 | N | RI | 1 | 0 | 0 | N | N | 2 | 30 | 51 | NON-ERD |
| CC95-71 | 1971 | 1904 | 67 | M |  |  | <10 | 10+ |  | PHENOBARB., PHENYTOIN | UK | N | N | NO | N | N | 1380 | NO HS | N | NO | 2 | 0 | 1 | N | N | NA |  | 27 | NON-ERD |
| CC8-72 | 1972 | 1900 | 72 | M | Y |  | >40 | 0-2 | WC | NO DETAIL | UK | N | N | NO | N | N | 1387 | NO HS | N | NO | 4 | 1 | 0 | N | N | 1 |  | 50 | NON-ERD |
| CC23-72 | 1972 | 1908 | 65 | M | Y |  | <10 | 10+ | MC | PHENOBARB. | TBI | N | Y | HI | Y | N | 1290 | NO HS | N | CI | 4 | 1 | 2 | Y | Y | 0.5 | 41 | 50 | NON-ERD |
| CC24-72 | 1972 | 1901 | 72 | M | Y |  | 20-40 | 10+ | WC | PHENYTOIN | UK | N | Y | NO | N | N | 1400 | NO HS | Y | CI | 7 | 2 | 1 | N | N | 2 |  | 50 | NON-ERD |
| CC134-72 | 1972 | 1889 | 83 | F | Y | Y |  | 10+ | MC | NO DETAIL | HS | N | Y | INJ | N | Y | 1186 | TYPE 2/3 | N | CI | 3 | 1 |  | N | N | 2 |  | 40 | SUDEP (POSS) |
| CC249-72 | 1972 | 1895 | 79 | F | Y |  |  |  |  | PHENOBARB. | UK | N | N | NO | N | N | 1154 | NO HS | N | NO | 2 | 1 | 0 | Y | N | NA |  | 50 | NON-ERD |
| CC152-73 | 1973 | 1901 | 72 | F | Y | Y | <10 | 10+ | MC | NO DETAIL | PI | N | Y | NO | N | Y | 1219 | TYPE 1 | N | PI | 3 | 2 | 0 | N | N | 3 |  | 46 | NON-ERD |
| CC232-73 | 1973 | 1936 | 36 | M | Y |  | <10 | 10+ | PC | PHENYTOIN | TBI | Y | Y | HI | Y | N | 1444 | NO HS | N | NO | 1 | 1 | 0 | N | N | 0.5 |  | 49 | NON-ERD |
| CC578-73 | 1973 | 1920 | 63 | F | Y |  | <10 | 10+ | PC | PHENOBARB. | HS | N | N | HI | Y | N | 1137 | TYPE 1 | N | NO | 3 | 1 | 0 | Y | N | NA |  | 49 | NON-ERD |
| CC326-74 | 1974 | 1924 | 50 | M | Y | Y | >40 | 2to10 | MC | PHENOBARB., AND OTHERS | UK | Y | N | NO | N | N | 1380 | NO HS | N | AI | 2 | 0 | 0 | N | N | NA |  |  | ERD |
| CC112-75 | 1975 | 1887 | 87 | M |  |  | 20-40 |  |  | NO DETAIL | TBI | N | N | NO | Y | N | 1233 | NO HS | N | CI | 1 | 0 | 0 | Y | N | 1 | 51 | 47 | NON-ERD |
| CC202-75 | 1975 | 1896 | 73 | F | Y |  | >40 | 2to10 |  | NO DETAIL | ND/AD | Y | Y | INJ | N | N | 896 | NO HS | N | NO | 1 | 0 | 0 | Y | N | 0 | 30 | 47 | NON-ERD |
| CC97-76 | 1976 | 1929 | 46 | F | Y |  |  |  |  | NO DETAIL | MCD | N | N | NO | N | Y | 1205 | NO HS | Y | NO | 3 | 0 | 1 | Y | N |  |  | 48 | ERD |
| CC184-76 | 1976 | 1914 | 62 | F | Y |  | 20-40 | 0-2 |  | NO DETAIL | O/S | Y | N | NO | N | N | 1219 | NO HS | N | CI | 2 | 1 | 0 | Y | Y | NA |  |  | NON-ERD |
| CC195-76 | 1976 | 1935 | 41 | M | Y |  | 20-40 | 0-2 |  | PHENOBARB., PHENYTOIN | UK | N | N | INJ | N | N | 1380 | NO HS | N | CI | 2 | 1 | 0 | N | N | 1 |  | 46 | NON-ERD |
| CC171-77 | 1977 | 1905 | 72 | F | Y |  | >40 | 2to10 |  | NO DETAIL | ND/AD | Y | Y | INJ | N | N | 912 | NO HS | N | CI | 3 | 0 | 0 | Y | N | 2 | 110 | 45 | NON-ERD |
| CC391-77 | 1977 | 1906 | 71 | F | Y | Y | <20 | 10+ | MC | NO DETAIL | UK | N | Y | INJ | N | N | 1022 | TYPE 1 | N | NO | 2 | 1 | 1 | Y | N | 1 |  | 45 | NON-ERD |
| CC83-78 | 1978 | 1904 | 74 | M | Y |  | <10 | 10+ | PC | PHENOBARB., PHENYTOIN | HS | N | Y | INJ | N | N | 1250 | BILAT/ASYM | N | NO | 3 | 0 | 1 | N | N | 1 |  | 44 | NON-ERD |
| CC103-78 | 1978 | 1923 | 55 | M | Y | Y | <20 | 10+ | MC |  | UK | Y | Y | NO | N | N | 1362 | NO HS | N | CI | 4 | 2 | 1 | Y | N |  | 117 | 44 | NON-ERD |
| CC114-78 | 1978 | 1921 | 57 | M | Y | Y | <20 | 10+ | MC | PHENYTOIN, CARB | UK | Y | Y | HI | Y | N | 1222 | NO HS | N | NO | 2 | 1 | 0 | Y | N |  | 150 | 44 | NON-ERD |
| CC115-78 | 1978 | 1903 | 73 | M | Y |  | 20-40 | 10+ | WC | PHENOBARB., PHENYTOIN | UK | N | Y | NO | Y | N | 1324 | NO HS | N | NO | 3 | 2 | 1 | Y | N |  |  | 44 | NON-ERD |
| CC121-78 | 1978 | 1915 | 63 | M |  |  | <20 | 10+ |  | NO DETAIL | HS | N | Y | NO | N | N | 1410 | TYPE 1 | N | CI | 7 | 2 | 1 | Y | Y | 2 |  | 44 | NON-ERD |
| CC47-79 | 1979 | 1942 | 36 | M |  |  | 20-40 | 10+ | WC |  | TBI | Y | N | HI | Y | N | 1366 | TYPE 1 | N | NO | 2 | 1 | 0 | N | N | 1 | 26 | 43 | NON-ERD |
| CC86-79 | 1979 | 1932 | 46 | M | Y |  | 20-40 | 10+ | MC | NO DETAIL | MCD | N | N | NO | N | N | 1433 | NO HS | Y | NO | 2 | 1 | 0 | Y | N | 1 | 60 | 43 | NON-ERD |
| CC162-79 | 1979 | 1917 | 62 | F |  |  |  |  | WC |  | MCD | N | Y | NO | N | Y | 1472 | NO HS | Y | CI | 3 | 1 | 0 | Y |  | 3 | 32 | 43 | NON-ERD |
| CC164-79 | 1979 | 1902 | 77 | F | Y | Y | 20-40 | 10+ | MC | PRIMIDONE, PHENOBARB. | UK | N | Y | NO | N | N | 975 | NO HS | N | CI | 1 | 1 | 0 | Y | N | NA |  | 43 | NON-ERD |
| CC165-79 | 1979 | 1944 | 35 | F |  |  | <10 | 10+ | PC | PHENYTOIN, CARB, VALP., CLONAZ. | MCD | Y | Y | HI | Y | Y | 1469 | NO HS | Y | NO | 1 | 0 | 0 | Y | N | 1 |  | 43 | SUDEP (POSS) |
| CC235-79 | 1979 | 1915 | 62 | M | Y |  | >40 | 0-2 | MC |  | CVD | Y | N | INJ | N | N | 1157 | NO HS | N | RI | 3 | 2 | 0 | N | N | 1 | 34 | 43 | NON-ERD |
| CC249-79 | 1979 | 1902 | 77 | M |  |  | >40 | 10+ | WC | NO DETAIL | UK | Y | Y | NO | N | N | 964 | NO HS | N | CI | 4 | 1 | 0 | Y | N | 1 | 27 | 43 | NON-ERD |
| CC252-79 | 1979 | 1906 | 73 | M | Y |  | >40 | 0-2 | WC | NO DETAIL | ND/AD | Y | N | INJ | N | N | 1320 | BILAT/ASYM | N | RI | 3 | 1 | 0 | Y | N | 4 | 40 | 43 | NON-ERD |
| CC25-80 | 1980 | 1909 | 70 | M |  |  | <10 | 10+ | MC |  | HS | N | Y | NO | N | N | 1178 | TYPE 1 | N | CI | 1 | 0 | 0 | Y | N | 4 |  | 42 | NON-ERD |
| CC40-80 | 1980 | 1891 | 89 | M | Y | Y |  | 10+ | MC | PHENOBARB., PHENYTOIN | MCD | N | Y | INJ | N | N | 1097 | NO HS | Y | CI | 2 | 1 | 0 | Y | N |  | 90 | 42 | NON-ERD |
| CC119-80 | 1980 | 1904 | 77 | F | Y | Y | <10 | 10+ | WC | PARALDEHYDE, PHENOBARB. | PI | N | Y | INJ | N | Y | 1028 | NO HS | N | PI | 4 | 1 | 0 | Y | N | 1 | 85 | 42 | NON-ERD |
| CC127-80 | 1980 | 1912 | 68 | M |  |  | <10 | 10+ |  | PHENOBARB., PHENYTOIN | MCD | N | Y | NO | N | N | 960 | NO HS | Y | CI | 2 | 0 | 0 | Y | N | 4 |  | 42 | NON-ERD |
| CC128-80 | 1980 | 1930 | 50 | F | Y |  | <10 | 10+ | PC | PHENOBARB., PHENYTOIN | UK | N | Y | NO | N | N | 1210 | NO HS | N | NO | 4 | 2 | 0 | N | N | 2 |  | 42 | NON-ERD |
| CC153-80 | 1980 | 1926 | 54 | M | Y |  | >40 | 2to10 | MC | PHENYTOIN | CVD | N | Y | NO | N | N | 1378 | NO HS | N | CI | 2 | 1 | 0 | Y | N | 3 |  | 45 | NON-ERD |
| CC167-80 | 1980 | 1905 | 85 | F | Y |  | >40 | 0-2 |  | PHENOBARB. | ND/AD | Y | N | NO | Y | N | 1143 | NO HS | N | RI | 1 | 1 | 0 | N | N | 1 | 34 | 42 | NON-ERD |
| CC246-80 | 1980 | 1937 | 43 | M |  | Y | <10 | 10+ | MC | PHENOBARB., PHENYTOIN, OTHERS | PI | N | Y | INJ | N | N | 1136 | NO HS | N | RI | 3 | 1 | 0 | Y | N |  |  | 42 | NON-ERD |
| CC269-80 | 1980 | 1937 | 43 | M | Y | Y | <20 | 10+ | MC |  | UK | Y | Y | HI | Y | N | 1292 | NO HS | N | NO | 5 | 2 | 1 | Y | N | 3 | 56 | 42 | SUDEP (POSS) |
| CC292-80 | 1980 | 1923 | 57 | M |  |  | <20 | 10+ |  | NO DETAIL | O/S | N | N | NO | N | N | 1010 | NO HS | N | CI | 3 | 1 | 1 | Y | Y |  |  | 42 |  |
| CC327-80 | 1980 | 1906 | 74 | F | Y | Y | <20 | 10+ | WC | PHENYTOIN, PRIMIDONE, PHENOBARB. | UK | N | Y | NO | N | N | 1150 | NO HS | N | NO | 1 | 0 | 1 | N | N | 1 |  | 42 | NON-ERD |
| CC335-80 | 1980 | 1903 | 77 | F | Y | Y | >40 | 2to10 |  | PHENOBARB., PHENYTOIN | ND/AD | Y | Y | NO | N | N | 915 | NO HS | N | NO | 2 | 1 | 0 | Y | N | 2 |  | 42 | NON-ERD |
| CC341-80 | 1980 | 1922 | 58 | F | Y | Y | <10 | 10+ | PC | PHENOBARB., PHENYTOIN, OTHERS | HS | N | Y | NO | N | N | 1130 | BILAT/ASYM | N | NO | 3 | 1 | 0 | Y | N |  |  | 42 | NON-ERD |
| CC20-81 | 1981 | 1914 | 67 | M | Y | Y | <10 | 10+ | WC | PHENOBARB., PHENYTOIN | HS | N | Y | NO | N | N | 1511 | BILAT | N | CI | 4 | 2 | 0 | N | N | NO DATA |  | 41 | NON-ERD |
| CC95-81 | 1981 | 1920 | 61 | M | Y | Y | <10 | 10+ | MC | NO DETAIL | HS | N | Y | NO | N | N | 1349 | BILAT/ASYM | N | CI | 4 | 2 | 1 | Y | Y | 3 |  | 41 | NON-ERD |
| CC163-81 | 1981 | 1933 | 48 | F | Y |  | 20-40 | 10+ |  | PHENOBARB., PHENYTOIN | HS | N | N | INJ | N | N | 1020 | BILAT/ASYM | N | NO | 9 | 2 | 1 | Y | N |  |  | 36 | NON-ERD |
| CC191-81 | 1981 | 1902 | 79 | M | 0 | Y | <10 | 10+ | MC | PHENYTOIN | HS | N | Y | NO | Y | N | 1265 | TYPE 1 | N | NO | 3 | 2 | 0 | Y | N | 3 |  | 41 | NON-ERD |
| CC275-81 | 1981 | 1942 | 39 | M | Y | Y | <20 | 10+ | PC | NO DETAIL | UK | N | Y | INJ | N | N | 1355 | NO HS | N | NO | 3 | 2 | 0 | Y | N | 1 |  | 41 | NON-ERD |
| CC276-81 | 1981 | 1956 | 25 | F | Y | Y | <10 | 10+ | PC | NO DETAIL | HS | N | Y | HI | Y | N | 1337 | TYPE 1 | N | NO | 2 | 1 | 0 | Y | Y | 2 | 55 | 41 | SUDEP (POSS) |
| CC144-82 | 1982 | 1912 | 70 | F | Y | Y | <10 | 10+ | MC | NO DETAIL | HS | N | Y | NO | N | N | 1360 | TYPE 1 | N | NO | 3 | 2 | 1 | Y | N | 2 |  | 40 | NON-ERD |
| CC194-82 | 1982 | 1918 | 64 | M | Y |  | <10 | 10+ | WC | NO DETAIL | HS | N | Y | NO | Y | Y | 1403 | TYPE 2/3 | N | NO | 4 | 1 | 1 | N | N | 7 |  | 40 | NON-ERD |
| CC13-83 | 1983 | 1934 | 51 | F |  |  | >40 | 0-2 |  | NO DETAIL | O/S | N | N | NO | N | N | 1442 | NO HS | N | RI | 3 | 1 | 1 | Y | Y | 2 | 370 | 39 | NON-ERD |
| CC210-83 | 1983 | 1899 | 85 | F |  |  | >40 | 10+ |  | NO DETAIL | UK | N | N | NO | N | N | 1010 | NO HS | N | CI | 2 | 1 | 0 | Y | N |  |  | 39 | NON-ERD |
| cc269-83 | 1983 | 1905 | 78 | F | Y |  | <20 | 10+ | WC | PHENOBARB., PHENYTOIN | UK | N | Y | INJ | N | N | 1150 | NO HS | N |  | 1 | 1 | 0 | N | N | 4 |  | 39 | NON-ERD |
| CC135-84 | 1984 | 1916 | 67 | F | Y |  | >40 | 10+ |  | VALPRROATE | ND/AD | Y | Y | NO | N | N | 1081 | NO HS | N | NO | 4 | 2 | 0 | N | N | NA |  | 38 | NON-ERD |
| CC38-85 | 1985 | 1933 | 52 | F | Y | N | <20 | 10+ |  | PHENYTOIN,PRIMIDONE, VALP. | UK | N | Y | INJ | N | N | 1100 | NO HS | N | NO | 2 | 1 |  | Y | N | 3 |  | 37 | SUDEP (POSS) |
| CC65-85 | 1985 | 1902 | 83 | M | Y | Y |  |  |  | PHENOBARB., PHENYTOIN | HS | N | Y | NO | N | N | 1150 | TYPE 1 | N | NO | 4 | 1 | 0 | Y | Y | 5 | 100 | 37 | NON-ERD |
| CC9-86 | 1986 | 1924 | 62 | M | Y |  | <20 | 10+ | PC | PHENOBARB., PHENYTOIN, CARB | UK | 0 | Y | HI | Y | N | 1070 | NO HS | N | NO | 4 | 1 |  | N | N | 2 |  | 32 | SUDEP (POSS) |
| CC21-86 | 1986 | 1929 | 57 | M | Y |  | >40 | 10+ | PC | PHENOBARB., PHENYTOIN | O/S | Y | N | NO | N | N | 1390 | TYPE 1 | N | NO | 4 | 1 | 0 | N | N | 1 |  |  |  |
| CC79-86 | 1986 | 1925 | 61 | M | Y |  | <20 | 10+ |  | PHENOBARB., PHENYTOIN | UK | N | Y | HI | Y | N | 1420 | NO HS | N | NO | 4 | 1 | 0 | Y | N | 1 |  | 36 | SUDEP (POSS) |
| CC85-86 | 1986 | 1919 | 68 | F | Y |  | >40 | 2to10 | PC | NO DETAIL | UK | N | N | NO | N | N | 982 | NO HS | N | CI | 4 | 0 | 2 | Y | N | 0 | 62 | 36 | NON-ERD |
| CC38-87 | 1987 | 1925 | 62 | F | Y |  | >40 | 2to10 |  | PHENYTOIN | UK | N | N | INJ | N | N | 1403 | NO HS | N | NO | 2 | 1 | 0 | N | N |  |  | 35 | NON-ERD |
| CC41-87 | 1987 | 1911 | 76 | F |  |  |  | 10+ |  | PHENYTOIN | UK | Y | N | NO | N | N | 1118 | NO HS | N | NO | 7 | 3 | 0 | Y | N | 3 | 180 | 35 | NON-ERD |
| CC48-87 | 1987 | 1950 | 37 | M | Y | Y | <10 | 10+ | MC | VALP., CARB | UK | N | Y | NO | N | N | 1400 | NO HS | N | NO | 3 | 1 | 0 | Y | Y | 3 | 120 | 35 | SUDEP (POSS) |
| CC56-87 | 1987 | 1916 | 71 | M | Y |  | >40 | 10+ |  | PHENOBARB. | TBI | N | N | INJ | N | N | 1008 | NO HS | N | NO | 4 | 1 | 1 | N | N | 1 |  | 35 | NON-ERD |
| CC28-88 | 1988 | 1946 | 42 | M | Y | Y | 20-40 | 10+ | WC | NO DETAIL | TBI | N | N | HI | Y | N | 1632 | NO HS | N | NO | 3 | 2 | 1 | Y | N | 0 | 58 | 44 | SUDEP (POSS) |
| CC41-88 | 1988 | 1900 | 88 | F | Y | Y | <20 | 10+ |  | PHENOBARB., PHENYTOIN | HS | N | Y | NO | N | N | 1016 | TYPE 1 | N | CI | 4 | 2 | 1 | Y | N | 2 |  | 34 | SUDEP (POSS) |
| cc50-88 | 1988 | 1944 | 44 | F | Y |  |  | 0-2 |  |  | UK | N | Y | HI | Y | N | 1060 | NO HS | N | CI | 4 | 2 | 0 | N | N | 2 | 52 | 34 | SUDEP (POSS) |
| CC89-88 | 1988 | 1930 | 58 | M |  |  |  |  | MC |  | UK | N | Y | INJ | N | N | 1600 | NO HS | N | NO | 4 | 2 | 1 | Y | N | 3 | 400 | 34 | SUDEP (POSS) |
| CC71-91 | 1991 | 1932 | 59 | M |  |  |  | 10+ |  | PHENOBARB., PHENYTOIN | PI | N | Y | HI | Y | N | 1147 | NO HS | N | PI | 6 | 1 | 1 | N | N | 1 |  | 31 | NON-ERD |
| CC19-92 | 1992 | 1962 | 30 | M |  |  | <10 | 10+ | MC |  | PI | Y | Y | NO | N | Y | 702 | BILAT | N | PI | 1 | 0 | 0 | N | N | 0 | 220 | 30 | SUDEP (POSS) |
| CC20-92 | 1992 | 1937 | 54 | F | Y |  | <20 | 10+ | MC | PHENYTOIN, CARB | UK | N | N | INJ | N | N | 1227 | NO HS | N | NO | 2 | 1 | 0 | Y | N | 1 | 1000 | 30 | SUDEP (POSS) |
| CC40-92 | 1992 | 1932 | 58 | F |  |  | <10 | 10+ | WC | PHENYTOIN | O/S | N | Y | NO | N | N | 1072 | NO HS | N | NO | 7 | 4 | 1 | Y | N | 2 | 360 | 30 | NON-ERD |
| CC48-92 | 1992 | 1930 | 62 | F | Y |  | 20-40 | 10+ | MC | CARB | UK | Y | Y | HI | Y | N | 1102 | NO HS | N | NO | 9 | 0 | 1 | Y | N | 4 |  | 30 | NON-ERD |
| CC61-92 | 1992 | 1933 | 59 | M | Y |  | >40 | 0-2 | MC |  | CVD | Y | Y | INJ | N | N | 1013 | NO HS | N | CI | 5 | 1 | 0 | Y | Y | 4 |  | 30 | NON-ERD |
| CC13-93 | 1993 | 1946 | 46 | F |  |  | <10 | 10+ | WC | NO DETAIL | MCD | N | N | NO | N | Y | 890 | BILAT | Y | NO | 6 | 2 | 1 | Y | N | 2 | 500 | 29 | SUDEP (POSS) |
| CC23-93 | 1993 | 1927 | 66 | M | Y |  | >40 | 0-2 | WC | PHENYTOIN | UK | N | N | NO | N | N | 1460 | NO HS | N | NO | 5 | 4 | 0 | N | N | 1 |  | 30 | NON-ERD |
| CC37-93 | 1993 | 1925 | 68 | M | Y |  | >40 | 10+ | WC | PHENYTOIN | UK | N | N | NO | N | N | 1275 | NO HS | N | NO | 3 | 1 | 0 | N | N | 0 |  | 29 | NON-ERD |
| CC61-93 | 1993 | 1925 | 67 | M | Y |  | >40 | 0-2 |  | NO DETAIL | CVD | N | N | NO | N | N | 1279 | NO HS | N | AI | 3 | 1 | 1 | N | Y | 4 | 40 | 29 | NON-ERD |
| CC60-94 | 1994 | 1933 | 61 | M | Y |  | >40 | 10+ | WC | PHENYTOIN, CARB | UK | Y | N | NO | N | N | 1200 | NO HS | N | NO | 2 | 1 | 0 | Y | N | 3 |  | 28 | NON-ERD |
| CC44-95 | 1995 | 1928 | 65 | M | Y |  | >40 | 2to10 | WC | NO DETAIL | TBI | N | N | HI | Y | N | 1338 | TYPE 2/3 | N | NO | 6 | 2 | 1 | Y | N | 3 | 129 | 27 | NON-ERD |
| CC34-96 | 1996 | 1928 | 67 | M |  | Y | >40 | 10+ |  | NO DETAIL | UK | Y | N | NO | N | N | 1260 | NO HS | N | NO | 2 | 1 | 1 | Y | Y | 4 |  | 26 | NON-ERD |
| CC73-96 | 1996 | 1927 | 69 | M | Y |  | >40 | 0-2 | MC | NO DETAIL | UK | N | N | NO | N | N | 1540 | NO HS | N | NO | 4 | 1 | 1 | Y | N | 2 |  | 28 | NON-ERD |

**Supplemental Table 1. Case details of epilepsy history, pathology, stains conducted and post-mortem data**.

Abbreviations. **Aetiology of epilepsy** : UK= unclear, PI =early/perinatal infarct/event, MCD = malformation of cortical development, TBI = traumatic brain injury, HS = hippocampal sclerosis, CVD = cerebrovascular disease, ND/AD=neurodegenerative disease , O/S =other structural lesion. **Injuries** : INJ = reported injury with seizures, HI = Head injury history (whether reported with seizure or not), NO= no reports or positive information of injury . **Hippocampal sclerosis (HS) diagnosis** : No hippocampal sclerosis,, TYPE 1 = unilateral ILAE type 1 HS, TYPE 2/3= unilateral ILAE HS type 2/3, BILAT = bilateral (TYPE 1 HS), BILAT/ASYM = bilateral asymmetrical sclerosis. **Cerebrovascular Disease (CVD)** : No clear CVD =NO, Neonatal Infarct (Inc. Ulegyria) = PI, Small vessel disease and lacunar infarcts = CI, Old regional infarct or stroke = RI, Acute infarcts, Ischaemic Necrosis, Haemorrhage = AI. **Cause of death** : Possible SUDEP = SUDEP (POSS), Other Epilepsy Related Death = ERD, Non-Epilepsy Related Death = NON-ERD. **Antiseizure medications** : Phenobarb = phenobarbitone, Carb = carbamazepine. **Seizure frequency and control** : episodes of poor control, status epilepticus, reports of seizure clusters = PC, well controlled rare seizures =WC, moderately well controlled = MC, no clear data on frequency blank. **Seizure duration and onset** are grouped and categorised as detailed in the text. N= Not confirmed/done, Y= Yes confirmed/done.
