# Supplementary material for: The prevalence of chronic traumatic encephalopathy in a historical epilepsy post‐mortem collection
Source: Brain Pathol. 2024 Nov 11;35(3):e13317. doi: 10.1111/bpa.13317 (PMC11961211; doi:10.1111/bpa.13317)
Supplement: Supplementary file 3 — Table S2. Summary of likely underlying aetiologies for epilepsy following evaluation of clinical and pathology records. In some cases, more than one pathology was present, including malformations with more than one malformation type. In 51 cases, a focal, presumed epileptogenic pathology was identified. In 20 cases, a focal pathology was assumed as a secondary and acquired pathology based on time course of epilepsy. [file BPA-35-e13317-s002.docx]

| **Likely Aetiology of Epilepsy** |
| --- |
| **Brain malformation n=8**  Polymicrogyria (2)  Heterotopia (2)  FCD1  Agenesis of corpus callosum + lipoma  Megalencephaly  Porencephaly  Dandy-Walker malformation + hydrocephalus |
| **Perinatal (early) infarct n = 6**  Ulegyric pattern n=5  Cortical infarct n=1 |
| **Hippocampal sclerosis (including TLE-HS) n= 19**  Unilateral ILAE HS type 1 n=11  HS type 2/3 n=2  Bilateral HS =6 |
| **Cerebrovascular disease n=6**  Old cortical infarct (2)  Small vessel disease + lacunar infarcts (2)  Acute/subacute ischaemia/haemorrhage (1) |
| **Other structural (focal lesion / dual pathologies) n=6**  Inflammatory pathology (3)  HS + infarct (1)  Old cystic scar (1)  Old abscess (1) |
| **Traumatic brain injury**  Post-traumatic epilepsy  **n=6** |
| **Neurodegenerative disease**  **AD-NC**  **n=7** |
| **Unknown (unlikely symptomatic/lesional epilepsy)**  **n= 44** |

Supplemental Table 2. Summary of likely underlying aetiologies for epilepsy following evaluation of clinical and pathology records. In some cases, more than one pathology was present, including malformations with more than one malformation type. In 51 cases a focal, presumed epileptogenic pathology was identified. In 20 cases a focal pathology was assumed as a secondary and acquired pathology based on time course of epilepsy.
