# Supplementary material for: The prevalence of chronic traumatic encephalopathy in a historical epilepsy post‐mortem collection
Source: Brain Pathol. 2024 Nov 11;35(3):e13317. doi: 10.1111/bpa.13317 (PMC11961211; doi:10.1111/bpa.13317)
Supplement: Supplementary file 4 — Table S3. Multivariate logistic regression analysis for glial tau pathology in relation to clinical factors with significant factors shown in bold red and trends in red. Exp(B) = Odds ratio and 95% confidence interval (CI) shown. [file BPA-35-e13317-s004.docx]

|  |  | GLIAL TAU / ARTAG (ANY REGION) | | | | GRANULAR TAU AGGREGATES | | | |
| --- | --- | --- | --- | --- | --- | --- | --- | --- | --- |
|  |  |  |  |  |  |  |  |  |  |
|  |  | Sig. | Exp(B) | 95% C.I.for EXP(B) | | Sig. | Exp(B) | 95% C.I.for EXP(B) | |
|  |  |  |  | Lower | Upper |  |  | Lower | Upper |
|  | HISTORY OF /cognitive decline | 0.601 | 0.69 | 0.172 | 2.773 | 0.261 | 2.394 | 0.523 | 10.97 |
|  | AGE AT DEATH | 0.762 | 1.008 | 0.956 | 1.064 | 0.408 | 0.976 | 0.92 | 1.035 |
|  | GENDER MALE | 0.02 | 5.335 | 1.303 | 21.847 | 0.014 | 0.122 | 0.023 | 0.656 |
|  | AGE OF ONSET OF EPILEPSY | 0.371 | 0.982 | 0.943 | 1.022 | 0.063 | 1.045 | 0.998 | 1.095 |
|  | RESIDENT AT INSTITUTE OR EPILEPSY CENTRE | 0.285 | 2.384 | 0.485 | 11.724 | 0.188 | 0.303 | 0.051 | 1.792 |
|  | DEVELOPMENTAL/LEARNING DELAY | 0.664 | 0.572 | 0.046 | 7.116 | 0.956 | 1.08 | 0.071 | 16.484 |
|  | TBI PRESENT | 0.079 | 3.678 | 0.86 | 15.739 | 0.052 | 0.196 | 0.038 | 1.012 |
|  | SUSPECTED SUDEP | 0.521 | 0.513 | 0.067 | 3.95 | 0.242 | 4.536 | 0.361 | 57.018 |

Supplemental Table 3. Multivariate logistic regression analysis for glial tau pathology in relation to clinical factors with significant factors shown in bold red and trends in red. Exp(B) = Odds ratio and 95% confidence interval (CI) shown.
