# Supplementary material for: The prevalence of chronic traumatic encephalopathy in a historical epilepsy post‐mortem collection
Source: Brain Pathol. 2024 Nov 11;35(3):e13317. doi: 10.1111/bpa.13317 (PMC11961211; doi:10.1111/bpa.13317)
Supplement: Supplementary file 5 — Table S4. Comparison of clinical and tissue protocol variables in epilepsy cases with negligible pTau present to cases with pTau. Logistic regression analysis statistics shown for variables and 95% confidence intervals (CI), odds ratio and significant values in red. [file BPA-35-e13317-s005.docx]

|  | **No neuronal/glial AT8 N=9**  **Mean (range) or % cases** | **AT8 present**  **N=93**  **Mean (range) or % cases** | **Significance, p value** | **OR** | **95% CI** | |
| --- | --- | --- | --- | --- | --- | --- |
| **Age at death (years)** | 49.4 (26-70) | 62.82 (25-89) | .015 | .941 | .895 | .988 |
| **Age onset of epilepsy (years)** | 13.7 (1-33) | 32.1 (1-84) | .111 | .955 | .902 | 1.011 |
| **Duration of epilepsy (years)** | 24.2 (9-48) | 27.1 (0.1-71) | .773 | .993 | .950 | 1.039 |
| **% with poor seizure control** | 40% | 28.6% | .593 | 1.667 | .257 | 10.823 |
| **% with TBI** | 33% | 21.5% | .423 | .548 | .126 | 2.387 |
| **% with epileptogenic brain lesion** | 44% | 51.1% | .704 | 1.306 | .329 | 5.173 |
| **Number of blocks examined (range)** | 2.5 (1-4) | 3.1 (1-9) | .285 | .755 | .451 | 1.264 |
| **Hippocampal block number (range)** | 0.89 (0-2) | 1.12 (0-4) | .418 | .679 | .267 | 1.730 |
| **PMI (days)** | 1.6 (0.5-4) | 2 (0.5-7) | .435 | .796 | .448 | 1.412 |
| **Fixation time (days)** | 47.28 | 134.8 | .186 | .981 | .953 | 1.009 |
| **FFPE time (years)** | 41 (26-57) | 48 (34-57) | .025 | 1.131 | 1.016 | 1.259 |

**Supplemental Table 4. Comparison of clinical and tissue protocol variables in epilepsy cases with negligible pTau present to cases with pTau.** Logistic regression analysis statistics shown for variables and 95% confidence intervals (CI), Odds ratio and significant values in red.
