# Supplementary material for: The prevalence of chronic traumatic encephalopathy in a historical epilepsy post‐mortem collection
Source: Brain Pathol. 2024 Nov 11;35(3):e13317. doi: 10.1111/bpa.13317 (PMC11961211; doi:10.1111/bpa.13317)
Supplement: Supplementary file 6 — Data S1. Supporting information. [file BPA-35-e13317-s003.docx]

Supplemental Methods

| **Tau and other antibodies** | **Source, Clone** | **Clonality** | **Method** | **Dilution** | **Pre-treatments, details** |
| --- | --- | --- | --- | --- | --- |
| AT8 (Ser202, Thr205) | 90206, Autogen Bioclear | Mouse, Monoclonal | Bond-Max system (Leica Microsystems) | 1 : 1200 | Bond Labelled Streptavidin-Biotin Detection System, Automated IHC - Epitope Retrieval ER1 Solution, DAB incubation time 5 minutes |
| CP13 (Ser 202) | Kind gift from the Feinstein Institutes for Medical Research; developed by Peter Davies | Mouse, Monoclonal | Ventana Discovery autostainer (Roche) | 1:200 | Detection performed with Cy5 kit (  07551215001); epitope retrieval: CC1, 24min, 91^o^C  (06414575001) |
| AT100 (Thr212, Ser 214) | MN1060, Invitrogen | Mouse, Monoclonal | Ventana Discovery autostainer (Roche) | 1:500 | Detection performed with Rhodamine 6G kit (  07988168001); epitope retrieval: CC1, 24min, 91^o^C  (06414575001) |
| AT180 (Thr231) | MN1040, Invitrogen | Mouse, Monoclonal | Ventana Discovery autostainer (Roche) | 1:500 | Detection performed with Rhodamine 6G kit (  07988168001); epitope retrieval: CC1, 24min, 91^o^C  (06414575001) |
| PHF1 (Ser396, Ser404) | Kind gift from the Feinstein Institutes for Medical Research; developed by Peter Davies | Mouse, Monoclonal | Ventana Discovery autostainer (Roche) | 1:2000 | Detection performed with Cy5 kit (  07551215001) epitope retrieval: ULTRA CC2, 24min, 91^o^C  (05424542001) |
| AT8 (IF) | MN1020, Invitrogen, 1:1200, | Mouse, Monoclonal | Ventana Discovery autostainer (Roche) | 1:1200 | Detection performed with FAM kit (  07988150001) epitope retrieval: ULTRA CC2, 24min, 91^o^C  (  05424542001) |
| Amyloid beta | 6F/3D (Dako) |  | Ventana Discovery Autostainer | 1 : 50 | Detection performed with ChromoMap DAB kit  (05266645001); epitope retrieval:  15 minutes formic acid treatment and CC1 (24min, 91^o^C, (06414575001) |
| AT8/GFAP  GFAP  AT8 | Z0334, Dako  MN1020, Invitrogen, 1:1200, | Rabbit, Polyclonal  Mouse, Monoclonal | Ventana Discovery Autostainer | 1 : 2500  1:1200 | See text below for detail of double labelling chromogenic protocol and reagents |

Table. List of antibodies used in the study.

**AT8 single labelling protocol**

Paraffin blocks were cut into sections at a thickness of 5 μm using a microtome. Sections were left to air dry overnight, and then allowed to bake in a 45-50 ºC oven overnight. Automated IHC was run via the Bond-Max system (Leica Microsystems) using primary antibody anti-AT8 (1:1200, Innogenetics, AutogenBioclear). Each staining run included positive controls from a confirmed AD case. Any sections that were damaged or detached from slides following the first protocol were repeated a minimum of three times, and if automated staining continued to produce inadequate staining, sections were immunostained manually using the same anti-AT8 (1:1200, Innogenetics, AutogenBioclear). For this method, Sections were deparaffinised and rehydrated in xylene (2 x 5 min), followed by ethanol in decreasing concentrations of 100% (2 x 3 min) to 70% (1 x 3 min). Following a rinse, sections were then submerged in 1% hydrogen peroxide (15 min) to quench endogenous peroxidases, rinsed, then immersed in a Pyrex dish containing Antigen Unmasking Solution, Citric Acid Based (1:1000, Vector, H-3300 tris buffer, low pH) and microwaved at 800W for 12 min for epitope retrieval. Sections were then washed in PBS (10 min on shaker), circled using a PAP pen, and blocked in 2.5% Normal Horse Serum (Vector, S-2012; 20 min). Sections were incubated at room temperature in primary antibody diluted in Flex Antibody Diluent (Dako, EnVision, DM830) for an hour, washed in PBS (3 x 5 min on shaker), then incubated at room temperature in secondary antibody HRP Rabbit/Mouse (Dako, REAL, EnVision, K5007; 30 min). Following another wash in PBS (3 x 5 min on shaker), sections were developed using DAB+ Chromogen (X 50) (1:50, Dako, Real, K3468) diluted in Substrate Buffer (Dako, Real, K3468), and incubated until desired colour was achieved Sections were then rinsed in water and counterstained with Haematoxylin (1 x 10 s), rinsed, then submerged in acidic alcohol (1 x 2 s), and rinsed. Dehydration was done via ethanol in increasing concentrations of 70% (1 x 3 min) to 100% (2 x 3 min) and xylene (2 x 5 min), before mounting and cover-slipping in an automated cover-slipper (Leica Microsystems CV5030).

**GFAP-AT8 double labelling method**

For this method, the epitopes were labelled sequentially with conditions optimized in single chromogenic staining. The protocol for GFAP, applied in the first sequence, included antigen retrieval with Protease 1 (20min, 37^o^C, 05266688001, Roche), incubation with anti-rabbit GFAP polyclonal antibody (Z0334, Dako, 1h at room temperature, 1:2500), further incubation with anti-rabbit HRP (OmniMap Anti-Rabbit HRP, 05269679001) and DISCOVERY Purple detection kit (07053983001, Roche). Staining for AT8 was applied in the second staining sequence with antigen retrieval using CC2 (91^o^C, 24min, 05279798001, Roche), incubation with monoclonal anti-mouse AT8 antibody (MN1020, Invitrogen, 1:1200, 1h at RT), further incubation with anti-mouse HRP (OmniMap Anti-Mouse HRP, 05269652001, Roche) and epitope detection with ChromoMap DAB kit (05266645001, Roche). The slides were counterstained with hematoxylin (Hematoxylin II, 05277965001, Roche).

**Immunofluorescence method for pTau epitopes**

Single immunofluorescence labelling protocols were developed for each of the five antibodies against tau phosphorylated isoforms. The optimum epitope retrieval conditions and primary antibody concentrations were established per marker as follows: AT8 (ULTRA CC2, 24min, 91^o^C; 1:1200), AT100 (CC1, 24min, 91^o^C; 1:500), AT180 (CC1, 24min, 91^o^C; 1:500), CP13 (CC1, 24min, 91^o^C; 1:200) and PHF1 (ULTRA CC2, 24min, 91^o^C; 1:2000). The epitopes were then labelled with one of the following tyramide-amplified fluorophores: Cy5 (07551215001, Roche), Rhodamine 6G (07988168001, Roche) or FAM (07988150001, Roche) and counterstained with DAPI nuclear marker (QD DAPI, 05268826001, Roche). The stained slides were scanned with S60 Hamamatsu Fluorescence Whole Slide scanner with the following fluorescence filters: Alexa 568, Alexa 647, FITC and DAPI with the most optimal exposures per fluorophore.
